# Interpretable recurrent neural network models for dynamic prediction of the extubation failure risk in patients with invasive mechanical ventilation in the intensive care unit

**DOI:** 10.1186/s13040-022-00309-7

**Published:** 2022-09-27

**Authors:** Zhixuan Zeng, Xianming Tang, Yang Liu, Zhengkun He, Xun Gong

**Affiliations:** 1 Department of Emergency Medicine, The Second Xiangya Hospital of Central South University, Changsha, China; 2 Department of Rehabilitation, The Second Xiangya Hospital of Central South University, Changsha, China; 3 School of Computer Science and Engineering, Central South University, Changsha, China

**Keywords:** Extubation failure, Invasive mechanical ventilation, Recurrent neural network, Dynamic prediction, Time series, Shapley additive explanations value

## Abstract

**Background:**

Clinical decision of extubation is a challenge in the treatment of patient with invasive mechanical ventilation (IMV), since existing extubation protocols are not capable of precisely predicting extubation failure (EF). This study aims to develop and validate interpretable recurrent neural network (RNN) models for dynamically predicting EF risk.

**Methods:**

A retrospective cohort study was conducted on IMV patients from the Medical Information Mart for Intensive Care IV (MIMIC-IV) database. Time series with a 4-h resolution were built for all included patients. Two types of RNN models, the long short-term memory (LSTM) and the gated recurrent unit (GRU), were developed. A stepwise logistic regression model was used to select key features for developing light-version RNN models. The RNN models were compared to other five non-temporal machine learning models. The Shapley additive explanations (SHAP) value was applied to explain the influence of the features on model prediction.

**Results:**

Of 8,599 included patients, 2,609 had EF (30.3%). The area under receiver operating characteristic curve (AUROC) of LSTM and GRU showed no statistical difference on the test set (0.828 vs. 0.829). The light-version RNN models based on the 26 features selected out of a total of 89 features showed comparable performance as their corresponding full-version models. Among the non-temporal models, only the random forest (RF) (AUROC: 0.820) and the extreme gradient boosting (XGB) model (AUROC: 0.823) were comparable to the RNN models, but their calibration was deviated.

**Conclusions:**

The RNN models have excellent predictive performance for predicting EF risk and have potential to become real-time assistant decision-making systems for extubation.

**Supplementary Information:**

The online version contains supplementary material available at 10.1186/s13040-022-00309-7.

## Background

Invasive mechanical ventilation (IMV) is the primary method of respiratory support for patients in the intensive care unit (ICU). The operation of removing endotracheal tube from the patient is referred to as extubation. For IMV patients, early successful extubation is expected by clinicians since prolonged IMV is associated with an increased incidence of complications such as ventilator-associated pneumonia and airway trauma [1], higher mortality [2–4] and more intensive care cost [5]. However, premature extubation may lead to extubation failure (EF), which also causes worse prognosis and higher cost [6, 7]. Thus, accurate decision-making of extubation is crucial for the treatment of patients with IMV.

Despite many clinical protocols and predictive methods have been developed, the decision-making of extubation remains challenging for clinicians. The spontaneous breathing trial (SBT) is the most widely used extubation protocol, which employs a T-tube trial or a low-level pressure support (≤ 8cmH_2_O) ventilation for 0.5–2 h [8]. However, studies showed that 13–29% of patients who passed a SBT still suffered from EF [9–11]. In addition, other measurements including the Rapid Shallow Breathing Index (RSBI) [12], cough strength [13], handgrip strength [14], dyspnea intensity and respiratory muscles ultrasound [15], were applied for predicting EF, but the results indicated that their accuracy was limited. In such a situation, a more precise prediction model is needed to assist clinicians to make the decision of extubation.

In recent years, machine learning models have shown potential to predict the EF risk in IMV patients. Kuo HJ et al. [16] and Hsieh MH et al. [17] built multilayer perceptron (MLP) neural network model for predicting the outcome of extubation among patients in ICU, and showed that MLP outperformed conventional predictors including RSBI, maximum inspiratory and expiratory pressure. Boosting models such as the light gradient boosting machine (LightGBM) [18] and the categorical boosting (CatBoost) model [19] also showed excellent performance for predicting EF. Besides, Fabregat A et al. [20] applied support vector machine (SVM), gradient boosting method (GBM) and linear discriminant analysis (LDA) for predicting the outcome of programmed extubation after SBT and found that SVM had best performance. However, the machine learning models developed in these studies only provided static predictions based on clinical data within a certain period (e.g., two hours [20], four hours [19] or longer) before extubation rather than dynamic predictions based on the time-series data throughout the duration of IMV. An innate limitation of these non-temporal models is that they are not designed for sequential data and incapable of continuously and dynamically predicting the EF risk for clinicians.

In this study, we report on development and validation of the recurrent neural network (RNN) models that can dynamically predict the EF risk at 4-h intervals for IMV patients. The predictive performance of the RNN models is compared to other non-temporal machine learning models. In addition, we explored the interpretability of our RNN models for a better understanding of their predictions.

## Methods

### Source of data

A retrospective cohort study was conducted on the IMV patients in a large critical-medicine database called the Medical Information Mart for Intensive Care IV (MIMIC-IV) [21]. The MIMIC-IV database provided comprehensive clinical records of the patients admitted to ICUs of the Beth Israel Deaconess Medical Center between 2008 and 2019. A local ethical review board (ERB) approval was achieved for building this database and all personal information was deidentified in accordance with the Health Insurance Portability and Accountability Act (HIPAA) standards, thus a ERB approval from our institution was exempted.

### Participants

All IMV patients who were extubated during their ICU stays were included. In the MIMIC-IV, direct records about the timestamp of intubation and extubation were not available. In order to determine the time span of IMV, we firstly judged the ventilation status of a patient according to the timestamped records of the oxygen delivery device and ventilator mode (Supplement Table 1). Then the start of IMV was determined when the ventilation status transferred to ‘Invasive ventilation’ from the other statuses, and the extubation was determined when a previous status of ‘Invasive ventilation’ was replaced by another status for a certain period (status of ‘Oxygen’ or ‘High flow oxygen’ for more than 2 h considering that a transient delivery of oxygen during invasive ventilation might represent SBT; and status of ‘None’ for more than 12 h considering that a transient ‘None’ status might occasionally happened due to record missing). The exclusion criteria were as follows: 1. not the first IMV of an included patient; 2. IMV less than 12 h; 3. age not between 16 and 89 years old; 4. more than 20% of the included features were missing, according to the definition of a missing feature for a single patient as the following: for static features, no observed value was retrieved in the database; for dynamic features, the observing frequency was less than 0.1 times per 4 h throughout the IMV process (see the following section about static and dynamic features). All eligible patients were included for model development and validation, without extra attempt to assess the appropriate sample size for this study. The outcome to be predicted was EF which was defined as the need for reintubation or noninvasive ventilation, or death within 48 h following extubation [8].

### Data collection and preprocessing

**Fig. 1:**
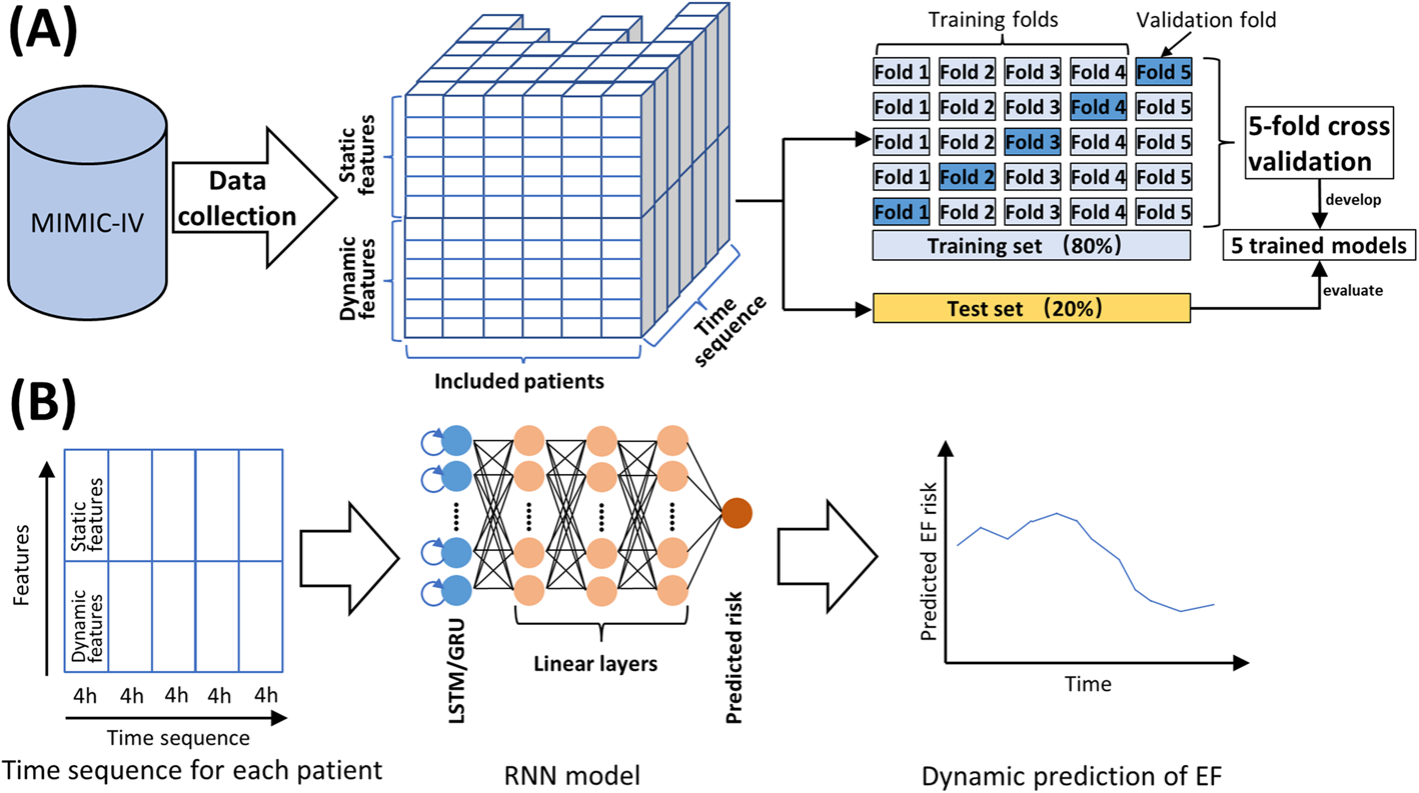
Study overview. **A** Raw data was collected from the MIMIC-IV, and preprocessed. Dynamic features varied over time, whereas static features kept constant. The patients had various lengths of time sequence. 80% of the included patients constituted the training set, and fivefold cross validation was used to optimize model hyperparameters and yield 5 trained models. The rest 20% constituted the test set for model validation. **B** The architecture of RNN contained one layer of LSTM/GRU neurons and three linear layers. RNN received a time sequence of feature vectors, and output a corresponding sequence of predicted EF risk. Abbreviations: EF extubation failure, GRU gated recurrent unit, LSTM long short-term memory, RNN recurrent neural network

Production of time series—Every included patient was represented by a sequence of feature vectors, with each feature vector containing the selected clinical features which were recorded within a time window of 4 h (Fig. 1A). We chose a 4-h window for a balance between continuously updating model predictions in real time and generating a manageable data size for our hardware.

Feature selection—We retrieved a comprehensive set of clinical features available in the MIMIC-IV database, including demographic characteristics, comorbidities, vital signs, Glasgow Coma Score (GCS), blood biochemistry, ventilator parameters, SBT, vasoactive drugs, use of antibiotics and sedatives, fluid balance, etc. These features were divided into static and dynamic features. Static features were relatively constant in the process of IMV, whereas dynamic features were temporally variant and routinely monitored daily or hourly. We also counted the average observing frequency of all dynamic features among the included patients, then excluded the features which were observed less than 0.1 times per 4 h since the records of these features were too sparse for building time series of a 4-h resolution (Supplement Table 2). The reserved dynamic features and the static features constituted the total feature set. In addition, we further selected a subset of the total feature set by applying a stepwise logistic regression model with both forward selection and backward elimination on the training set. This stepwise logistic regression model was developed based on the data in the last 4 h before extubation. The purpose was to build a light-version RNN based on fewer features and compare it to the full-version RNN.

Data preprocessing—For numeric features that were probably monitored more than once in a 4-h time window, the minimal, maximal, average value, standard deviation and delta value (difference from previous value) were calculated depending on the feature. Reasonable ranges were set for filtering out extreme values (Supplement Table 2). Observed values outside these ranges were treated as missing data. Categorical features were dummy encoded for representation. Missing values were imputed using the last observation carried forward [22]; missing values ahead of the first observation and missing values in a sequence without any observation were imputed using the mean of the training set. All numeric features were normalized by subtracting the mean and dividing by the variance, where the mean and the variance were derived from the training set. Besides, since the length of time sequence varied among different patients, batches of sequences were padded to a uniform length equivalent to the longest sequence, and were sequentially compacted into packed sequences as model input. This padding and packing manner enabled the model to handle variable-length sequences and to identify actual lengths of sequences without redundantly analyzing padding values for prediction.

### Development of RNN model

Recurrent neural network—RNN is a special artificial neural network designed for sequential data. RNN introduces cyclic paths to integrate past inputs and present inputs for prediction, just as owning a capability of ‘memory’. However, uncontrolled iterative cycles prevent RNN from preserving its cell state for a longer period, and tend to cause older inputs to exponentially influence the output, which leads to a common problem of vanishing or exploding gradient during model training. Gated units are introduced to mitigate these problems. The long short-term memory (LSTM) [23] unit, which contains three control gates and a memory cell, is a commonly used gated RNN unit. These three gates control the processes of updating cell state and producing output. The input gate decides whether to let inputs to change the current cell state; the forget gate decides whether to return the stored cell state to zero; the output gate decides whether to output the current cell state. In this manner, the LSTM model has longer ‘memory’ and avoids gradient problem, but it has much more parameters to be tuned than traditional RNN model, which results in a greater computing burden. The gated recurrent unit (GRU) [24] is a variant of the LSTM unit. It reduces three gates into two: the reset gate and the update gate, with less parameters and higher training efficiency. Conventionally, both the LSTM and the GRU read an input sequence in a forward direction (from past to present), which is referred to as unidirection. The bidirectional LSTM/GRU combines reading forward with reading backward for mining potential reverse temporal dependencies in a sequence. In this study, we only employed unidirectional LSTM and GRU for our task since a bidirectional model could not perform a backward reading using the future data in a real clinical scenario.

Model input and output—The architecture of our RNN models contained three linear layers which were sequentially connected to the LSTM/GRU layers. The linear layers shrunk the model output into one dimensional scalar between 0 and 1 as the predicted EF risk. When a trained RNN model received a time sequence of feature vectors, it could output a corresponding sequence of predicted EF risks (Fig. 1B). Assisted by the padding and packing technology, the RNN models were adapted to variable-length sequences, thus it was capable of updating prediction every 4 h as feature vectors accrued, based on a gradually extended sequence that contained all data from the start to the current timepoint.

Model training and validation—All included patients were split into the training set (80%) and the test set (20%). A five-fold cross-validation (CV) was applied on the training set to determine the optimal hyperparameters of our RNN models. Specifically, we applied a grid search strategy on all combinations of the following hyperparameter settings: hidden layers of LSTM/GRU (1, 2, 3, 4), hidden size (5, 10, 15, 20, 25), dropout (0, 0.25, 0.5, 0.75), learning rate (0.01, 0.001), activation function (sigmoid, ReLU). The combination of hyperparameters which had the highest average area under the receiver operating characteristic curve (AUROC) in the five-fold CV was the optimal setting, and the developed five models were selected for further validation on the test set (Fig. 1A). We chose a batch size of 32 for model iterative training and applied the Adam optimization algorithm for gradually modifying the model parameters. During model training, the cross-entropy loss function was used to assess how well the last predicted EF risk of a sequence fitted the real outcome (referred to as label: 0 for extubation success and 1 for extubation failure). The prior predicted risks were not included in the loss function since no corresponding outcome of extubation could be observed until the end of IMV. And so was the case with model validation on the test set, where the measures of model performance were evaluated based on the last prediction of the sequence.

In order to prevent information leakage of the test set during model development, we ensured that only the data of the training set was used in the following steps: building stepwise logistic regression model for feature selection, computing the mean and the variance for normalization of numeric features, and the five-fold CV. And the test set was reserved only for the final evaluation of model performance.

### Competitive non-temporal models

As mentioned before, non-temporal machine learning models, which were developed using clinical data within a certain period before extubation, were applied to predict EF risk in several previous studies. In this study, five respective non-temporal ML algorithms, including lasso logistic regression (lasso LR), SVM, MLP, random forest (RF) and extreme gradient boosting (XGB), were selected as baseline models. And the clinical data within the last 4 h before extubation was used for model development. Like the RNN models, five-fold CV on the training set was also applied to optimize hyperparameters of these non-temporal models and to develop five trained models for validation on the test set. Then their predictive performance was compared to the RNN models.

### Model interpretation

Artificial neural network was regarded as a black-box model since the impact of each feature on its prediction was hard to be assessed. This issue challenged its credibility, especially when such a model was applied in medical field. In our study, the Shapley additive explanations (SHAP) algorithm [25] was used to explain the contribution of each feature on a particular prediction produced by RNN model. Specifically, the prediction for an individual patient at any given time was approximately represented by the sum of the average predicted risk in our studied population and the SHAP values of all used features. In this manner, the SHAP value of each feature indicated how this feature influenced the prediction. A feature could drive the prediction towards either extubation failure (with positive SHAP value) or extubation success (with negative SHAP value). We applied a SHAP summary plot to visualize the global influence of all used features on the test set, and then provided a representative instance to show how the SHAP values explained the influence of features on the dynamic predictions for an individual IMV patient.

### Statistical analysis and measures of model performance

For both the training set and the test set, features between the successful and failed extubation group were compared using either Student t test, rank-sum test or Chi-square test as appropriate. Continuous features were described as mean (standard deviation) or median [interquartile range], and categorical features were described as number (percentage).

To evaluate the discriminative ability of our models, the AUROC was applied. DeLong’s test [26] was used to compare statistical difference between two AUROCs. The other measures included accuracy, F1 score (harmonic mean of precision and recall) [27] and the area under the precision-recall curve (AUPRC), where accuracy and F1 score were calculated under a threshold of 0.5. The statistical mean and 95% confidence interval for each measure were generated by aggregating the results of the five models developed from the five-fold CV. The model calibration was visualized by the calibration curve [28], which plotted means of decile-binned predicted probabilities versus corresponding means of actual probabilities in the patients in each bin. The calibration was assessed by inspecting the proximity between the calibration curve and the identity line of y = x which represented perfect calibration. Additionally, a decision curve analysis was used to evaluate the potential benefit of clinical decision-making based on predictions from the RNN models [29].

The RNN models were built using Pytorch version 1.7.1 (https://pytorch.org/get-started/previous-versions/#v171), and the non-temporal machine learning models were built using Scikit-learn package version 0.23.1 (https://scikit-learn.org/stable/install.html). Statistical analysis was performed using IBM SPSS Statistics software version 25.0. Two tailed *P* < 0.05 was considered as statistical significance.

## Results

### Participants and features

**Fig. 2:**
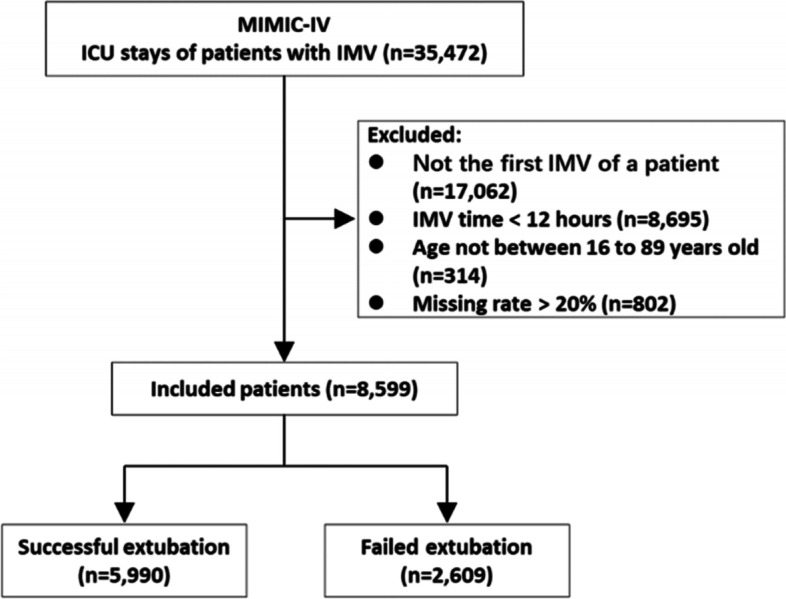
Flow chart of patient selection. Abbreviations: ICU intensive care unit, IMV invasive mechanical ventilation, MIMIC-IV Medical Information Mart for Intensive Care-IV

We ultimately included a total of 8,599 IMV patients in the MIMIC-IV database (Fig. 2), and 2609 patients suffered from EF (EF rate: 30.3%), due to reintubation (1392 patients), noninvasive ventilation (152 patients), and death (1065 patients) within 48 h following extubation. We randomly allocated 80% of the patients (6879 patients) to the training set and allocated the rest 20% (1720 patients) to the test set. EF rate was 30.4% (2089 patients) in the training set and 30.2% (520 patients) in the test set respectively. The longest ventilation time reached 919.0 h. All the included patients produced a total of 127,973 feature vectors in their time series. For feature selection, we originally retrieved a total of 101 features, of which 12 features had an average observing frequency less than 0.1 times per 4 h. Thus, the final total feature set contained 89 features (20 static features and 69 dynamic features) (Supplement Table 2). Comparisons of the baseline static features between the successful and failed extubation group were provided in Table 1. In addition, comparisons of the dynamic features was provided in Supplement Table 3. Considering each patient had a sequence of dynamic features, we used the data from the first 4-h window for comparison.

**Table 1:** Baseline static features between the successful and failed extubation group

	IMV patients (*n* = 8599)					
	Training set (*n* = 6879)		*P* value	Test set (*n* = 1720)		*P* value
	Successful extubation (*n* = 4790)	Failed extubation (*n* = 2089)		Successful extubation (*n* = 1200)	Failed extubation (*n* = 520)	
Gender (male), n (%)	2862 (59.7)	1228 (58.8)	0.470	680 (56.7)	292 (56.2)	0.885
Age (y, mean (SD))	63.07 (16.11)	63.18 (15.86)	0.811	63.69 (15.88)	64.29 (15.19)	0.464
BMI (kg/m2, mean (SD))	29.34 (7.52)	29.97 (8.60)	0.006	29.02 (7.62)	30.19 (9.22)	0.015
**Admission type**			< 0.001			< 0.001
Medical, n (%)	2927 (61.1)	1534 (73.4)		725 (60.4)	383 (73.6)	
Unscheduled surgical, n (%)	1619 (33.8)	511 (24.5)		414 (34.5)	131 (25.2)	
Scheduled surgical, n (%)	244 (5.1)	44 (2.1)		61 (5.1)	6 (1.2)	
**Comorbidities**						
Myocardial infarction, n (%)	932 (19.5)	397 (19.0)	0.686	234 (19.5)	94 (18.1)	0.533
Congestive heart failure, n (%)	1319 (27.5)	596 (28.5)	0.414	370 (30.8)	155 (29.8)	0.713
Chronic pulmonary disease, n (%)	1250 (26.1)	577 (27.6)	0.198	331 (27.6)	137 (26.3)	0.638
Peptic ulcer disease, n (%)	151 (3.2)	64 (3.1)	0.905	37 (3.1)	19 (3.7)	0.642
Liver disease, n (%)	671 (14.0)	453 (21.7)	< 0.001	157 (13.1)	111 (21.3)	< 0.001
Renal disease, n (%)	884 (18.5)	406 (19.4)	0.355	239 (19.9)	101 (19.4)	0.865
Peripheral vascular disease, n (%)	685 (14.3)	284 (13.6)	0.462	156 (13.0)	66 (12.7)	0.923
Cerebrovascular disease, n (%)	725 (15.1)	428 (20.5)	< 0.001	183 (15.2)	118 (22.7)	< 0.001
Paraplegia, n (%)	238 (5.0)	155 (7.4)	< 0.001	51 (4.3)	37 (7.1)	0.018
Diabetes, n (%)	1367 (28.5)	594 (28.4)	0.953	358 (29.8)	162 (31.2)	0.624
Malignant cancer, n (%)	513 (10.7)	300 (14.4)	< 0.001	135 (11.3)	55 (10.6)	0.745
Metastatic solid tumor, n (%)	206 (4.3)	147 (7.0)	< 0.001	52 (4.3)	23 (4.4)	0.964
AIDS, n (%)	27 (0.6)	16 (0.8)	0.417	5 (0.4)	3 (0.6)	0.950
Ventilation time (hours, median [IQR])	25.0 [17.0, 54.1]	47.0 [24.0, 103.0]	< 0.001	24.0 [17.2, 48.0]	47.7 [26.0, 103.0]	< 0.001

### Stepwise logistic regression model

The stepwise logistic regression model selected a total of 26 features as predictors. As shown in Table 2, medical admission, comorbidities of cerebrovascular disease, paraplegia and metastatic solid tumor, CRRT, higher values of heart rate, respiratory rate, PaCO2, sodium, anion gap, INR, Ppeak, PEEP, inspiratory flow rate and dose of norepinephrine were associated with increased risk of EF. Conversely, scheduled surgical admission, use of intravenous antibiotics, higher values of weight, SpO2, eyes and motor GCS, hemoglobin, and calcium were associated with decreased risk of EF.

**Table 2:** Feature selection of stepwise logistic regression model

Features	OR [95% CI]	*P* value
**Demographic characteristics**		
Weight (with each 10 kg increment)	0.971 [0.947, 0.995]	0.017
Admission type (unscheduled surgical as reference)		
Medical	1.195 [1.054, 1.356]	0.005
Scheduled surgical	0.696 [0.492, 0.986]	0.041
**Comorbidities**		
Cerebrovascular disease	1.395 [1.198, 1.626]	< 0.001
Paraplegia	1.685 [1.340, 2.118]	< 0.001
Metastatic solid tumor	1.587 [1.241, 2.029]	< 0.001
**Vital signs**		
HR (with every 10 beats/min increment)	1.047 [1.014, 1.082]	0.005
RR	1.019 [1.007, 1.031]	0.001
SpO2	0.943 [0.921, 0.964]	< 0.001
**GCS**		
GCS eyes	0.773 [0.723, 0.826]	< 0.001
GCS motor	0.844 [0.811, 0.879]	< 0.001
**Blood biochemistry**		
PaCO2 (with every 10 mmHg increment)	1.151 [1.076, 1.231]	< 0.001
Glucose	1.002 [1.001, 1.004]	< 0.001
Hemoglobin	0.910 [0.880, 0.940]	< 0.001
BUN	1.008 [1.005, 1.012]	< 0.001
Creatinine (with every 0.1 mg/dl increment)	0.986 [0.979, 0.994]	< 0.001
Sodium (with every 10 mmol/L increment)	1.397 [1.275, 1.532]	< 0.001
Calcium	0.883 [0.813, 0.959]	0.003
Anion gap	1.061 [1.042, 1.081]	< 0.001
PTT	1.005 [1.002, 1.008]	0.002
INR	1.182 [1.064, 1.314]	0.002
**Ventilator parameters**		
Ppeak	1.037 [1.027, 1.047]	< 0.001
PEEP	1.082 [1.057, 1.107]	< 0.001
Inspiratory flow rate	1.011 [1.008, 1.013]	< 0.001
Norepinephrine (with every 0.1 mcg/kg/min increment)	1.308 [1.228, 1.394]	< 0.001
Intravenous antibiotics	0.843 [0.743, 0.957]	0.008
CRRT	1.734 [1.319, 2.280]	< 0.001

*Abbreviations: CI* confidence interval*, CRRT* continuous renal replacement therapy*, GCS* Glasgow Coma Scale*, HR* heart rate*, INR* international normalized ratio*, OR* odds ratio*, PEEP* positive end expiratory pressure*, Ppeak* peak inspiratory pressure*, PTT* partial thromboplastin time*, RR* respiratory rate

### Model development and comparison

**Fig. 3:**
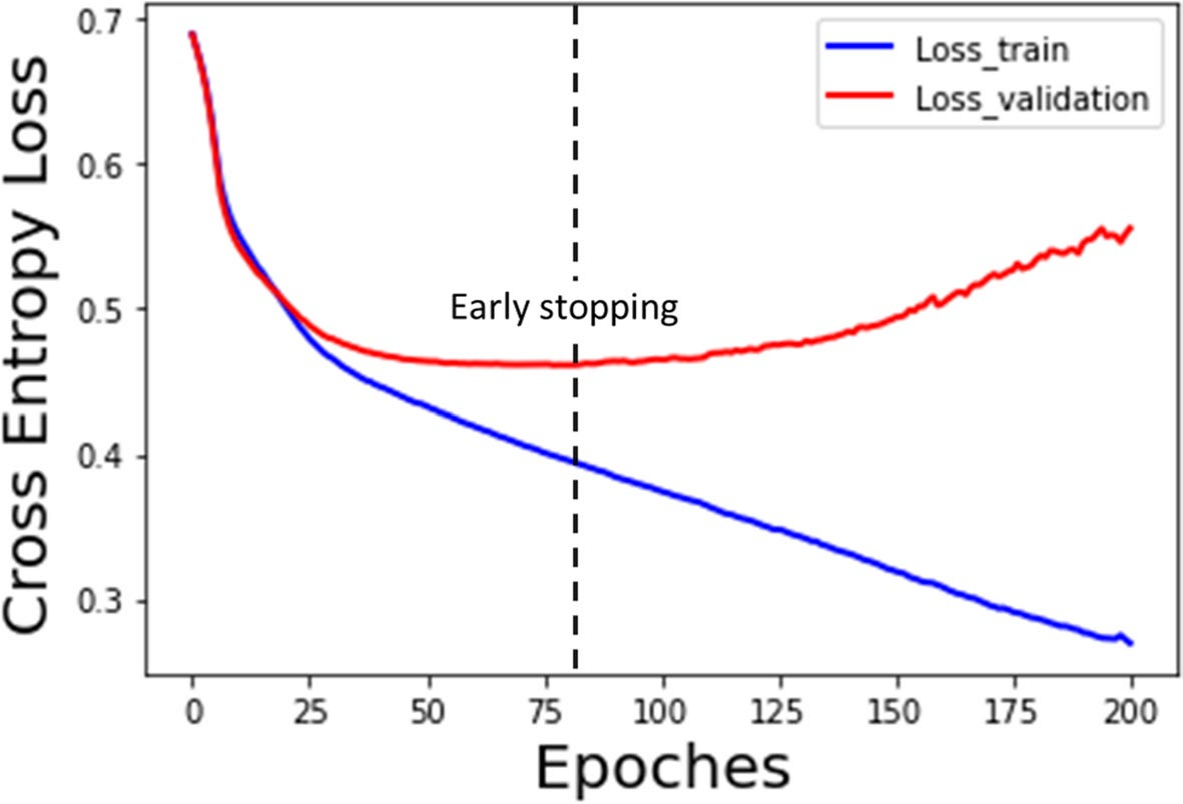
Instance of RNN training and early stopping. In each epoch, the model iteratively tuned its parameters by analyzing batches of sequences until reading through all sequences in the training set. As epochs increased, the cross-entropy loss in the training fold decreased gradually, whereas the loss in the validation fold firstly decreased and then increased. Early stopping was triggered if the loss in validation fold began to rise, in order to avoid over-fitting of the model

We trained two types of RNN models: LSTM and GRU, and five types of non-temporal machine learning models. Five-fold cross-validation was implemented for all these models on the training set, using time sequential data or the last four-hour data. In each fold cross-validation, the validation fold was used to early stop training. Figure 3 showed an instance of RNN training process. The optimized hyperparameters and the AUROCs of the five-fold CV for all the models were provided in Supplement Table 4. Both the LSTM and the GRU had an optimal architecture consisted of one layer of 20 LSTM/GRU neurons, with a learning rate of 0.001, a dropout rate of 0.5 and a ReLU activation function.

We assessed the predictive performance of our models on the test set by various measures. All the numeric measures were summarized in Table 3. In terms of comparing AUROCs, both LSTM and GRU model showed significantly higher AUROCs than lasso LR, SVM and MLP (p < 0.05 by DeLong’s test). When compared to RF and XGB, LSTM and GRU showed comparable AUROCs without statistical difference, with the most discrepant result between GRU and RF (0.829; 95% CI, 0.810 to 0.847 vs. 0.820; 95% CI, 0.801 to 0.838, P = 0.115). Additionally, we compared the light-version RNN models with their full versions. Our results demonstrated that the light version had comparable AUROC to the full version (LSTM vs. light_LSTM: 0.828 vs. 0.827, *P* = 0.891; GRU vs. light_GRU: 0.829 vs. 0.825, *P* = 0.360), and even had slightly higher AUPRC for light_LSTM and light_GRU. Due to reduced quantity of used features, the light-version RNN models were more efficient and convenient for clinicians.

**Table 3:** Predictive performance of the models on the test set

	Accuracy [95%CI]	F1 score [95%CI]	AUPRC [95%CI]	AUROC [95%CI]
LSTM	0.787 [0.766–0.806]	0.599 [0.585–0.612]	0.720 [0.714–0.726]	0.828 [0.809–0.846]
light_LSTM^a^	0.795 [0.790–0.800]	0.603 [0.585–0.621]	0.725 [0.718–0.732]	0.827 [0.809–0.845]
GRU	0.791 [0.777–0.804]	0.599 [0.560–0.639]	0.723 [0.713–0.733]	0.829 [0.810–0.847]
light_GRU^a^	0.791 [0.783–0.798]	0.585 [0.564–0.605]	0.724 [0.714–0.734]	0.825 [0.806–0.843]
Lasso LR	0.793 [0.789–0.798]	0.585 [0.573–0.597]	0.716 [0.709–0.722]	0.814 [0.795–0.832]
SVM	0.794 [0.788–0.801]	0.549 [0.533–0.566]	0.717 [0.711–0.722]	0.816 [0.797–0.834]
MLP	0.784 [0.776–0.792]	0.580 [0.547–0.613]	0.693 [0.682–0.705]	0.812 [0.792–0.830]
RF	0.790 [0.786–0.795]	0.514 [0.494–0.535]	0.726 [0.722–0.729]	0.820 [0.801–0.838]
XGB	0.790 [0.788–0.792]	0.595 [0.585–0.604]	0.724 [0.715–0.732]	0.823 [0.804–0.841]

*Abbreviations: CI* confidence interval,* GRU* gated recurrent unit,* LSTM* long short-term memory,* LR* logistic regression,* MLP* multi-layer perceptron,* RF* random forest,* SVM* support vector machine,* XGB* extreme gradient boosting

^a^The light-version RNN models based on the 26 features selected by the stepwise logistic regression model

**Fig. 4:**
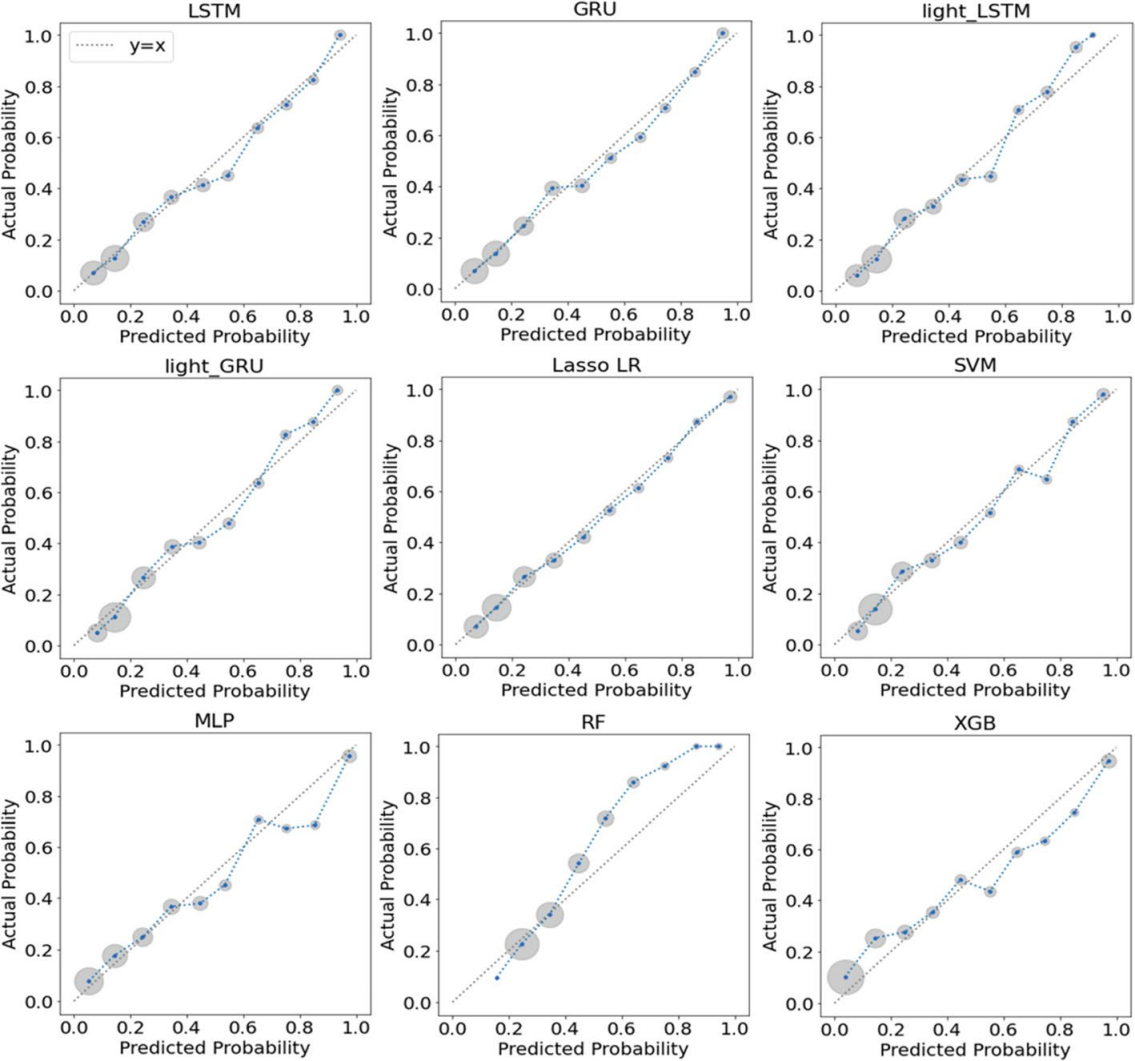
Calibration curves for model validation on the test set. For each model, the calibration curve plotted means of decile-binned predicted probabilities versus corresponding means of actual probabilities in the patients in each bin. As shown, each blue point of a calibration curve represented a bin and the size of the gray circle around represented the sample size of this bin. The dotted line was the identity line of y = x representing perfect calibration. Abbreviations: GRU gated recurrent unit, LSTM long short-term memory, LR logistic regression, MLP multi-layer perceptron, RF random forest, SVM support vector machine, XGB extreme gradient boosting

**Fig. 5:**
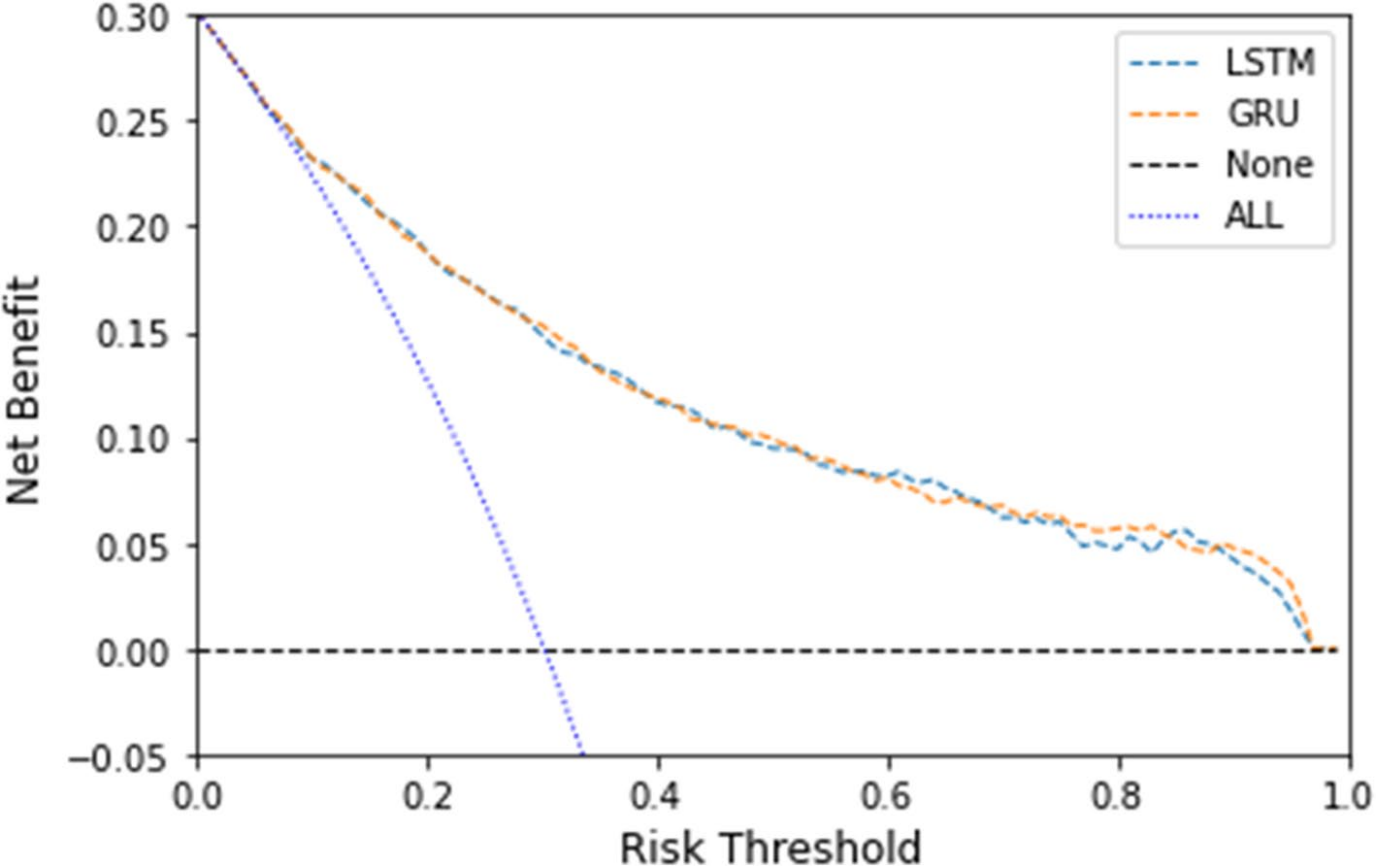
Decision curve analysis of the full-version RNN models on the test set. Abbreviations: GRU gated recurrent unit, LSTM long short-term memory

The calibration curves for our models on the test set were provided in Fig. 4. Overall, most models showed an excellent ability of calibration with their curves closely around the diagonal, especially GRU and Lasso LR. The calibration curves of MLP, RF and XGB deviated from the diagonal more obviously than the other models. RF underestimated the risk in high-risk bins, while MLP and XGB overestimated the risk in high-risk bins. Additionally, our study also provided a decision curve analysis for the full-version RNN models on the test set (Fig. 5).

### Interpretation of RNN models

**Fig. 6:**
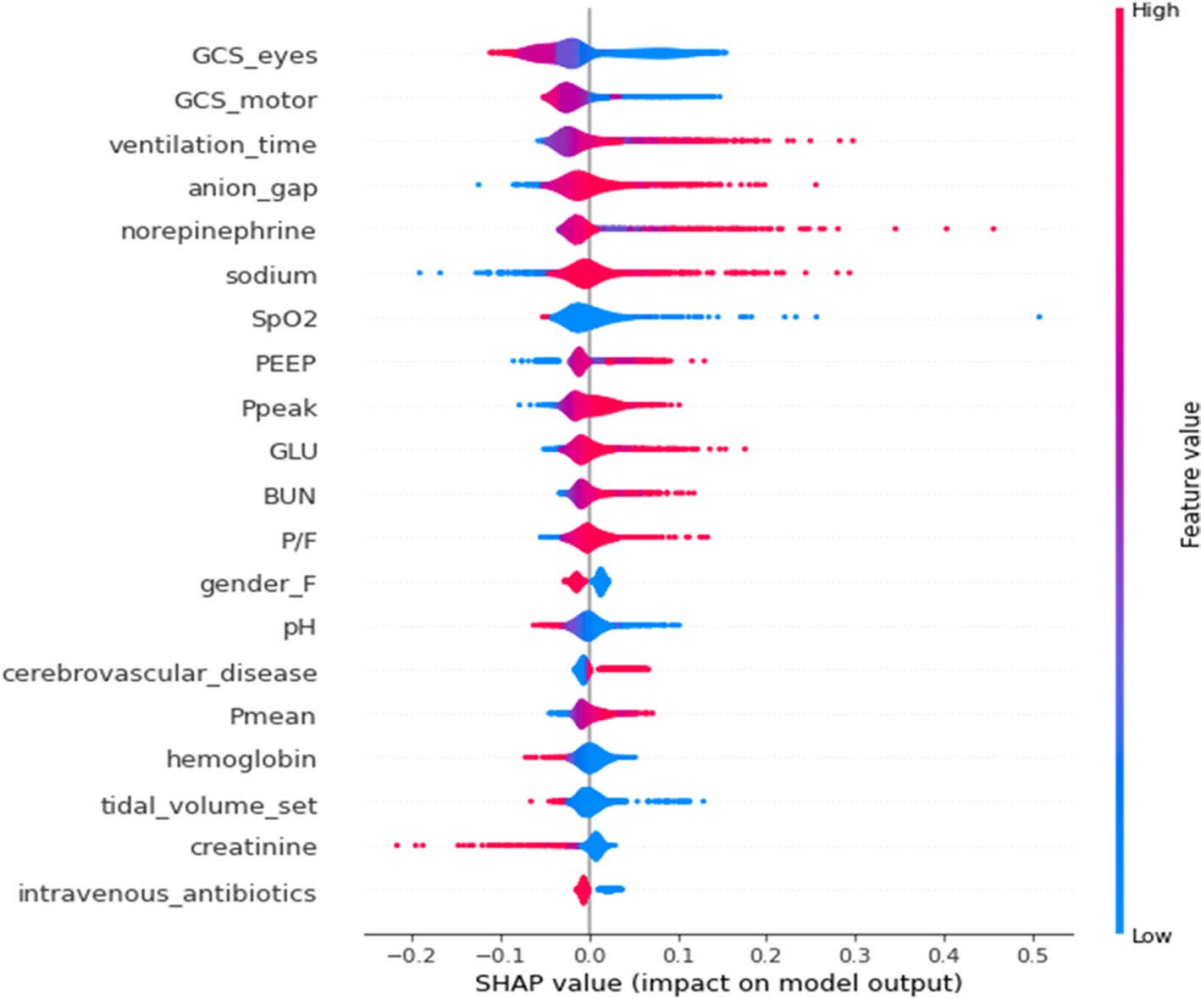
The impacts of the top 20 features on predictions of LSTM_1. Each colored dot of the plot demonstrated the impact of a feature on the prediction for a 4-h window in the time sequence of a patient. The color bar represented the feature value, from low (blue) to high (red). The x-axis indicated the impacts on the model output, driving the prediction towards extubation failure (positive SHAP value) or towards extubation success (negative SHAP value). Abbreviations: GLU glucose, PEEP positive end expiratory pressure, Pmean mean airway pressure, Ppeak peak inspiratory pressure, P/F PaO2/FiO2

We employed the SHAP value to evaluate the contribution of each feature in our RNN models. Figure 6 was a SHAP summary plot for one of the five developed LSTM models (referred to as LSTM_1). In Fig. 6, each colored dot of the plot demonstrated the impact of a feature on the prediction for a 4-h window in the time sequence of a patient, thus the entire plot summarized SHAP values for the predictions in all 4-h windows of all the patients in the test set. All the features were ranked according to their average explainable SHAP values and the top 20 features were showed. In addition, SHAP summary plots of the other four LSTM models were provided in the Supplement Fig. 1. In terms of evaluating the most impacting features, our results showed that 12 features (eyes GCS, motor GCS, SpO2, ventilation time, hemoglobin, sodium, anion gap, BUN, glucose, dose of norepinephrine, PEEP and gender_female) appeared in all the five SHAP summary plots, and 4 features (pH, Ppeak, tidal_volume_set and intravenous antibiotics) appeared in four of the five plots, indicating relatively consistent evaluations among the five LSTM models. In terms of impacting effect, lower value of eyes/motor GCS, SpO2, hemoglobin pH and tidal_volume_set drove the predictions towards EF, whereas longer ventilation time, higher ventilator parameters including PEEP and Ppeak, larger dose of norepinephrine and higher value of blood biochemistry including sodium, anion gap, glucose and BUN drove the predictions towards EF, and so did the absence of intravenous antibiotics. We also assessed SHAP values for GRU models, without obvious difference compared to LSTM.

**Fig. 7:**
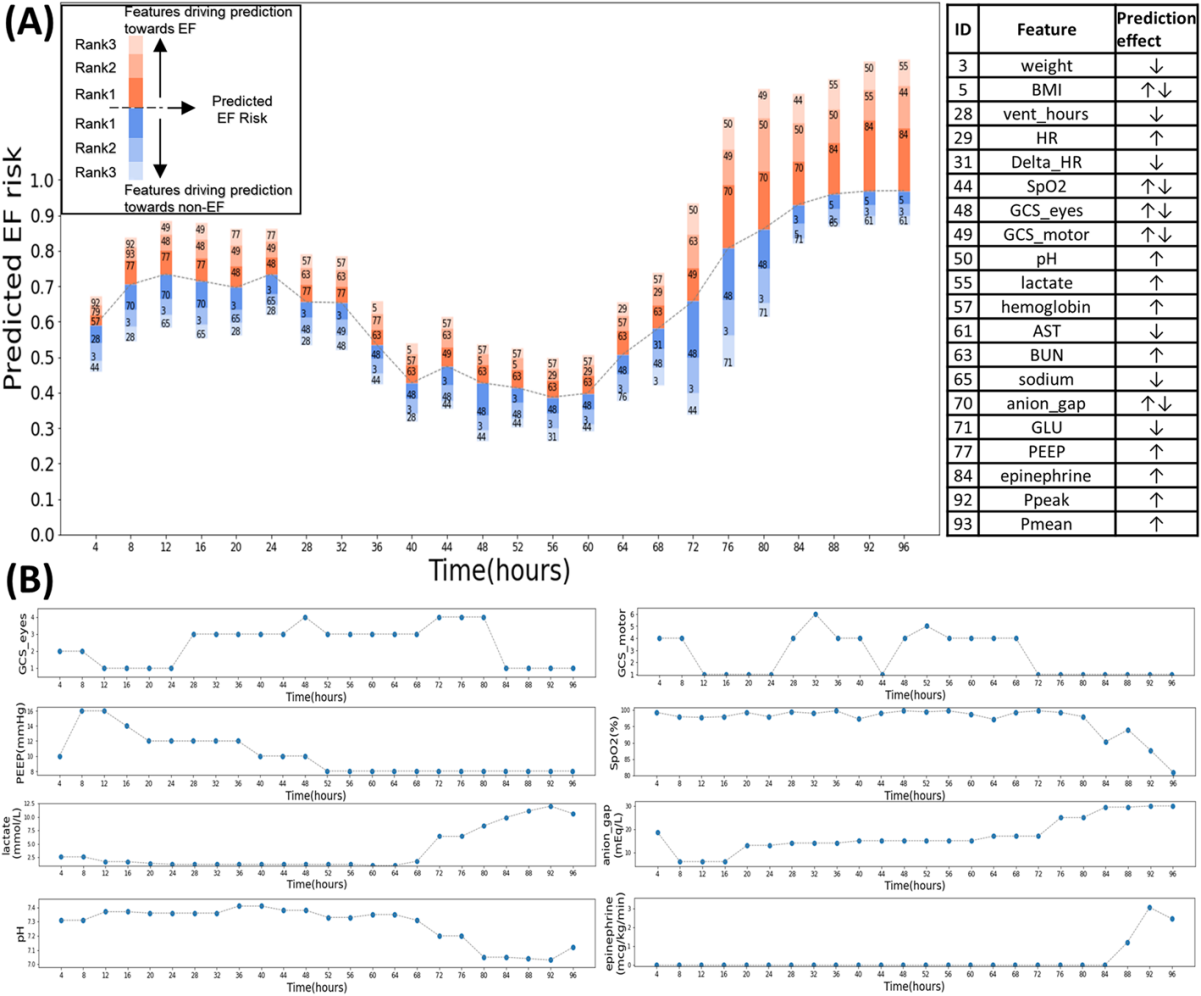
Dynamic predictions and SHAP values of a patient by LSTM_1. **A** The dotted line represented the dynamically predicted EF risk. The red bar with varied shadows above each prediction represented the top three features driving the prediction towards extubation failure, and the blue bar below each prediction represented the features driving the prediction towards extubation success. The index of feature ID used in the figure was provided in the table on the right. **B** Dynamic observations of the most important features for interpretation of the real-time predictions in (**A**)

At the individual level, we selected a representative patient from the test set and provided a dynamic analysis of the SHAP values throughout the IMV process for all the five LSTM models. The Fig. 7 showed our analysis for LSTM_1 and the Supplement Fig. 2 was for the other four LSTM models. This patient was a 50-year-old male with a comorbidity of liver disease. He was admitted to the medical ICU due to severe pneumonia and underwent a process of IMV for nearly 96 h, but he finally suffered EF due to death within following 48 h. As shown in Fig. 7A, LSTM_1 predicted this patient to have an EF risk above 60% during the first 24 h of IMV, since he did not have a full consciousness (low eyes/motor GCS) and needed a high level of PEEP (≥ 12 mmHg) for ventilative support (Fig. 7B). Then the patient’s condition improved as the needed PEEP decreased and eyes/motor GCS increased, thus the predicted risk decreased to about 40% during the next 36 h (the 24th hour to the 60th hour). We also noticed that the impact of eyes GCS on the prediction reversed as it increased (from ≤ 2 to ≥ 3) during this period. However, in the following duration of IMV, the prediction was drove towards EF again when this patient suffered from acidosis with elevated anion-gap and lactate, low SpO2 and even needed for epinephrine. In Supplement Fig. 2, the rest four LSTM models provided analogous trend of predictions, without obvious difference for the most impacting features driving their predictions. Specifically, all the LSTM models provided relatively high predicted risk (50–70%) during the first 24 h of IMV, and the low eyes/motor GCS and high PEEP were the most common risky features. Then these models downgraded EF risk until about the 64th hour, due to increased eyes/motor GCS and decreased PEEP, as seem in most plots. Finally, these models rapidly upgraded EF risk to about 90%, where the major risky features were low pH and SpO2, elevated anion-gap and lactate, need for epinephrine or norepinephrine, indicating acidosis, respiratory and circulatory failure.

## Discussion

In this study, we developed and validated interpretable RNN models for dynamically predicting EF risk of IMV patients in ICU. Our work was based on a retrospective study of time-series data of the eligible patients in the MIMIC-IV database. The developed RNN models could update their predictions at 4-h intervals until extubation, and their predictions were explained by the SHAP value that reflected the contribution of each used feature. Additionally, we also built light versions of our RNN models by reducing the employed features for convenience of clinical application. To our best knowledge, this is the first research to develop prediction model for dynamically predicting extubation risk.

Decision-making of extubation is a challenge in the treatment of IMV patient since EF seems inevitable even if clinicians take a large effort to exhaustively assess the patient. Traditional extubation protocols such as SBT and recently developed non-temporal machine learning prediction models can partly assist clinicians to make a confident decision. But these methods are limited in unscrambling the temporal dependance of various features and analyzing the tendency of patient’s condition, which makes them always provide a one-off prediction based on data obtained within a certain period. From the clinical perspective, it is undoubtedly more reasonable to regard the prediction of EF as a dynamical problem for which we need to take both the past and current situation of the patient into account. Such a meticulous clinical thinking is commendably reflected by the algorithm theory of RNN. The RNN model adapts to evolving clinical data during IMV and provides real-time guidance for extubation, which makes it more practical for clinicians than static predictive tools. Besides, continuously real-time prediction is potential to discover early opportunities of extubation, which are likely to be neglected by clinicians if they can only intermittently use static tools to confirm their empirical judgments for extubation.

In this study we evaluated a total of 89 features for model development. Such a total feature set was more comprehensive than most of the previous related researches. Some severity scores such as the Acute Physiology and Chronic Health Evaluation II (APACHE-II) and the Sequential Organ Failure Assessment (SOFA) score, which were employed as model features in previous studies [17, 18, 20], were not included in our study since our total feature set almost covered the constituents of these scores. Considering that these severity scores are calculated using a linear and additive combination of their constituents (e.g., APACHE-II is essentially a logistic regression model), we argue that the RNN is sophisticated enough to mine such a simple data pattern and it is redundant and inconvenient to repeatedly evaluate these scores at 4-h intervals. Besides, we applied the stepwise logistic regression model for further feature selection and developed light-version RNN models based on the selected 26 features. The stepwise logistic regression model is feasible to reduce features used in our RNN models, although itself is unable to analyze time-series data. Feature selection according to clinical consensuses or empirical advice is also considered for our study, but it is not objective and explorative as the data-based method.

As in all retrospective study on electronic health record, our extracted records had error data and missing data. We used reasonable ranges that were set by three experienced critical care physicians of our hospital to filter out error data. Then we imputed missing data using the last observation carried forward. Such an imputation method is considered to be biased since the condition of the patient constantly changes [30]. In order to reduce the potential imputation bias, we set an inclusion criterion of average observing frequency more than 0.1 times per 4 h for our features to avoid the situation in which an observed value would impute subsequently missing data for an overlong period. As a matter of fact, this imputation method is similar to the reality of clinical work where the clinician always carries the most recent detective value for analysis until a re-detection is considered necessary.

In this study we evaluated RNNs based on both LSTM and GRU units. Our result on the test set indicates that GRU is comparable to LSTM in terms of both discrimination and calibration. Both LSTM and GRU are commonly used for clinical predictive tasks based on time-series data [31–34]. Some previous researches selected a single type of RNN for their tasks without specific explanation [22, 32, 33, 35], but the truth is that no guideline about applying machine learning algorithms in clinical predictive tasks has been established. Despite that no significant difference was found, this study presented an overview of the two major types of RNNs for predicting EF risk. We also evaluated various non-temporal machine learning models. Considering that no unified time limitation of data collection was confirmed in previous researches, we selected the last 4 h that was in line with the time window for each feature vector used in our RNN models. The result suggests that none of the developed non-temporal models has improved performance compared to our RNN models, although several non-temporal models are excellent in terms of either discrimination or calibration. It is notable that no technique for prediction calibration (e.g., isotonic regression [36]) was applied for developing both the RNN and non-temporal models, ensuring that our result reflected the original calibration of these models.

We used the SHAP value to assess the impact of the features on model predictions. In terms of the global influence on the test set, most features provide expected contributions for predicting EF, such as an unclear consciousness (lower eyes and motor GCS), prolonged ventilation time, high ventilator parameters (PEEP, Ppeak), high dose of norepinephrine and low SpO2. Some features have rarely been studied or are considered to have ambiguous impacts on predictions. For instance, elevated anion gap drove prediction toward EF in our study, which was in line with a previous study that collected anion gap values of 127 intubated elderly patients on the day of extubation [36]. But another study including 61 patients suggested that low preextubation anion gap might indicate EF [37]. Elevated anion gap is clinically more common and often associates with metabolic acidosis caused by hypoxemia, shock, renal failure, etc., which may support elevated anion gap to be a risk factor of EF. We also see that high sodium and high glucose are top features that drive prediction toward EF. These two features are seldom reported and have not been treated as key factors for EF before, thus further research is needed to confirm their roles in EF.

We analyzed SHAP values for individual patient to show the dynamic changes of the top features impacting the real-time prediction. The representative instance presented in our study indicates that the LSTM models make prediction based on different leading features as the patient’s condition alters over time and some features can drive the prediction either towards EF or towards extubation success as their values change. The top features can reflect the most critical conditions of the patient currently, such as respiratory failure with high-parameter support of ventilator, circulative failure with vasoactive agents, or serious acidosis. In the other words, the most influential features will alter when a new crisis for the patient happens. As shown in Fig. 7, decreasing pH and elevating anion gap became the riskiest features after the 76th hour, but their influence went into decline at the 88th hour as this patient needed for epinephrine in the following 8 h, suggesting that the model considered this to be more critical than acidosis. Although the interpretation derived from SHAP values may not always be reasonable, it attempts to provide a transparent inspection into the prediction of the model and make the model more credible.

Our study has several limitations. Firstly, an objective limitation in this study is that only the last feature vector of a patient can be labeled since the outcome of extubation is retrospectively determinable only at the end of IMV. This differs from several previous RNN based studies which aim to dynamically predict whether some complications (e.g., death [22], acute kidney injury [31] or sepsis [35]) will happen within a following period of time. In this situation, our RNN models were trained according to the cross entropy between the last prediction and its label. Despite not being directly included in the loss function for model training, all the prior predictions are algorithmically responsible to yield the last prediction in our RNN models as the input sequence contains all the feature vectors. A large sample size (8,599 patients) can also partly compensate the reduced quantity of labeled feature vectors. Additionally, the advantage of using these variable-length sequences (12 to 919 h) is to train the RNN model to output a rational prediction at various timepoints during IMV, which is the foundation of model’s capability of dynamic prediction. Secondly, we excluded the patients with IMV less than 12 h. The purpose is to make our model to be more applicable for the patients undergoing difficult and prolonged extubation, since short IMV is more common in the routine postoperative patients who have a minimal risk of EF. But in the other hand, the model obtains no experience about predicting EF in the first 12 h of IMV. Thus, the predictive performance of our models may be weak in the early period of IMV. Thirdly, although we have included many features for model development, we can’t let our models to learn more about some novel techniques that seem valuable for our task, such as diaphragmatic function which can be measured by the endotracheal negative pressure in response to bilateral phrenic nerve stimulation [38] or by the diaphragm thickening fraction under ultrasound [39], lung ultrasound score [40], and cuff leak test [41]. The reason is that relative data is scarce in the MIMIC-IV database. In addition, the need for professional operation in these techniques may make our model inconvenient even if there is available data. Lastly, our study is a single-center retrospective study, thus our models may be effective for a limited population. Multiple-center prospective cohorts are needed for further model validation and model updating.

## Conclusions

In conclusion, we developed interpretable RNN models to dynamically predict EF risk in patients with IMV in this study. Our results demonstrated that the RNN models had excellent predictive performance, and the SHAP algorithm could assess the impact of each feature on the predictions of the RNN models. We argue that the RNN models have potential to be real-time assistant systems for decision-making of extubation, but a multiple-center prospective validation is needed before the RNN models can be applied in clinical practice.

## Supplementary Information

Additional file 1: SupplementFig 1. The impacts of the top 20 features on predictions of the other four LSTM models.

Additional file 2: SupplementFig 2. Dynamic predictions and SHAP values of a patient by the other four LSTM models.

Additional file 3: SupplementTable 1. Keywords of the oxygen delivery device and ventilator mode for different ventilation statuses.

Additional file 4: SupplementTable 2. Overview of the retrieved features.

Additional file 5: SupplementTable 3. Dynamic features between the successful and failed extubation group.

Additional file 6: SupplementTable 4. Optimized hyperparameters and AUROCs of the models in five-fold CV.

## Data Availability

Data of the MIMIC-IV is available on website at https://mimic-iv.mit.edu/. The extracted dataset used during the current study is available from the corresponding author on reasonable request.

## References

[1] Tobin MJ (1994). Mechanical ventilation. N Engl J Med.

[2] Peñuelas O, Frutos-Vivar F, Fernández C, Anzueto A, Epstein SK, Apezteguía C (2011). Characteristics and outcomes of ventilated patients according to time to liberation from mechanical ventilation. Am J Respir Crit Care Med.

[3] Bigatello LM, Stelfox HT, Berra L, Schmidt U, Gettings EM (2007). Outcome of patients undergoing prolonged mechanical ventilation after critical illness. Crit Care Med.

[4] Esteban A, Anzueto A, Frutos F, Alía I, Brochard L, Stewart TE (2002). Characteristics and outcomes in adult patients receiving mechanical ventilation: a 28-day international study. JAMA.

[5] Cooper LM, Linde-Zwirble WT (2004). Medicare intensive care unit use: analysis of incidence, cost, and payment. Crit Care Med.

[6] Thille AW, Harrois A, Schortgen F, Brun-Buisson C, Brochard L (2011). Outcomes of extubation failure in medical intensive care unit patients. Crit Care Med.

[7] Seymour CW, Martinez A, Christie JD, Fuchs BD (2004). The outcome of extubation failure in a community hospital intensive care unit: a cohort study. Crit Care.

[8] Boles JM, Bion J, Connors A, Herridge M, Marsh B, Melot C (2007). Weaning from mechanical ventilation. Eur Respir J.

[9] Frutos-Vivar F, Esteban A, Apezteguia C, González M, Arabi Y, Restrepo MI (2011). Outcome of reintubated patients after scheduled extubation. J Crit Care.

[10] Farias JA, Retta A, Alía I, Olazarri F, Esteban A, Golubicki A (2001). A comparison of two methods to perform a breathing trial before extubation in pediatric intensive care patients. Intensive Care Med.

[11] Esteban A, Alía I, Tobin MJ, Gil A, Gordo F, Vallverdú I (1999). Effect of spontaneous breathing trial duration on outcome of attempts to discontinue mechanical ventilation. Spanish Lung Failure Collaborative Group. Am J Respir Crit Care Med.

[12] Frutos-Vivar F, Ferguson ND, Esteban A, Epstein SK, Arabi Y, Apezteguía C (2006). Risk factors for extubation failure in patients following a successful spontaneous breathing trial. Chest.

[13] Duan J, Zhang X, Song J (2021). Predictive power of extubation failure diagnosed by cough strength: a systematic review and meta-analysis. Crit Care.

[14] Cottereau G, Messika J, Megarbane B, Guérin L, da Silva D, Bornstain C (2021). Handgrip strength to predict extubation outcome: a prospective multicenter trial. Ann Intensive Care.

[15] Dres M, Similowski T, Goligher EC, Pham T, Sergenyuk L, Telias I, et al. Dyspnea and respiratory muscles ultrasound to predict extubation failure. Eur Respir J. 2021;2100002. 10.1183/13993003.00002-2021 10.1183/13993003.00002-2021 33875492

[16] Kuo HJ, Chiu HW, Lee CN, Chen TT, Chang CC, Bien MY (2015). Improvement in the prediction of ventilator weaning outcomes by an artificial neural network in a Medical ICU. Respir Care.

[17] Hsieh MH, Hsieh MJ, Chen CM, Hsieh CC, Chao CM, Lai CC (2018). An Artificial Neural Network Model for Predicting Successful Extubation in Intensive Care Units. J Clin Med.

[18] Chen T, Xu J, Ying H, Chen X, Feng R, Fang X (2019). Prediction of extubation failure for intensive care unit patients using light gradient boosting machine. IEEE Access.

[19] Zhao QY, Wang H, Luo JC, Luo MH, Liu LP, Yu SJ (2021). Development and Validation of a Machine-Learning Model for Prediction of Extubation Failure in Intensive Care Units. Front Med (Lausanne).

[20] Fabregat A, Magret M, Ferré JA, Vernet A, Guasch N, Rodríguez A (2021). A machine learning decision-making tool for extubation in Intensive Care Unit patients. Comput Methods Programs Biomed.

[21] Goldberger AL, Amaral LA, Glass L, Hausdorff JM, Ivanov PC, Mark RG (2000). PhysioBank, PhysioToolkit, and PhysioNet: components of a new research resource for complex physiologic signals. Circulation.

[22] Thorsen-Meyer HC, Nielsen AB, Nielsen AP, Kaas-Hansen BS, Toft P, Schierbeck J (2020). Dynamic and explainable machine learning prediction of mortality in patients in the intensive care unit: a retrospective study of high-frequency data in electronic patient records. Lancet Digit Health.

[23] Hochreiter S, Schmidhuber J (1997). Long short-term memory. Neural Comput.

[24] Chung J, Gulcehre C, Cho K, et al. Empirical evaluation of gated recurrent neural networks on sequence modeling. arXiv Preprint. 2014. arXiv:1412.3555.

[25] Lundberg S, Lee S-I (2017). A unified approach to interpreting model predictions. Adv Neur In.

[26] DeLong ER, DeLong DM, Clarke-Pearson DL (1988). Comparing the areas under two or more correlated receiver operating characteristic curves: A nonparametric approach. Biometrics.

[27] Goutte C, Gaussier E, Losada DE, Fernández-Luna JM (2005). A probabilistic interpretation of precision, recall and F-score, with implication for evaluation. Advances in information retrieval.

[28] Alba AC, Agoritsas T, Walsh M, Hanna S, Iorio A, Devereaux PJ (2017). Discrimination and calibration of clinical prediction models: users' guides to the medical literature. JAMA.

[29] Vickers AJ, Elkin EB (2006). Decision curve analysis: a novel method for evaluating prediction models. Med Decis Making.

[30] Lachin JM (2016). Fallacies of last observation carried forward analyses. Clin Trials.

[31] Tomašev N, Glorot X, Rae JW, Zielinski M, Askham H, Saraiva A (2019). A clinically applicable approach to continuous prediction of future acute kidney injury. Nature.

[32] Van Steenkiste T, Ruyssinck J, De Baets L, Decruyenaere J, De Turck F, Ongenae F (2019). Accurate prediction of blood culture outcome in the intensive care unit using long short-term memory neural networks. Artif Intell Med.

[33] Yang Z, Tian Y, Zhou T, Zhu Y, Zhang P, Chen J (2021). Time-series deep survival prediction for hemodialysis patients using an attention-based Bi-GRU network. Comput Methods Programs Biomed.

[34] Meyer A, Zverinski D, Pfahringer B, Kempfert J, Kuehne T, Sündermann SH (2018). Machine learning for real-time prediction of complications in critical care: a retrospective study. Lancet Respir Med.

[35] Fagerström J, Bång M, Wilhelms D, Chew MS (2019). LiSep LSTM: A Machine Learning Algorithm for Early Detection of Septic Shock. Sci Rep.

[36] Suraseranivong R, Krairit O, Theerawit P, Sutherasan Y (2018). Association between age-related factors and extubation failure in elderly patients. PLoS One.

[37] Saugel B, Rakette P, Hapfelmeier A, Schultheiss C, Phillip V, Thies P (2012). Prediction of extubation failure in medical intensive care unit patients. J Crit Care.

[38] Dres M, Goligher EC, Heunks LMA, Brochard LJ (2017). Critical illness-associated diaphragm weakness. Intensive Care Med.

[39] DiNino E, Gartman EJ, Sethi JM, McCool FD (2014). Diaphragm ultrasound as a predictor of successful extubation from mechanical ventilation. Thorax.

[40] Soummer A, Perbet S, Brisson H, Arbelot C, Constantin JM, Lu Q (2012). Ultrasound assessment of lung aeration loss during a successful weaning trial predicts postextubation distress*. Crit Care Med.

[41] Kuriyama A, Jackson JL, Kamei J (2020). Performance of the cuff leak test in adults in predicting post-extubation airway complications: a systematic review and meta-analysis. Crit Care..

