# Supplementary material for: Interpretable recurrent neural network models for dynamic prediction of the extubation failure risk in patients with invasive mechanical ventilation in the intensive care unit
Source: BioData Min. 2022 Sep 27;15:21. doi: 10.1186/s13040-022-00309-7 (PMC9513908; doi:10.1186/s13040-022-00309-7)
Supplement: Supplementary file 1 — Additional file 1: SupplementFig 1. The impacts of the top 20 features on predictions of the other four LSTM models. [file 13040_2022_309_MOESM1_ESM.docx]

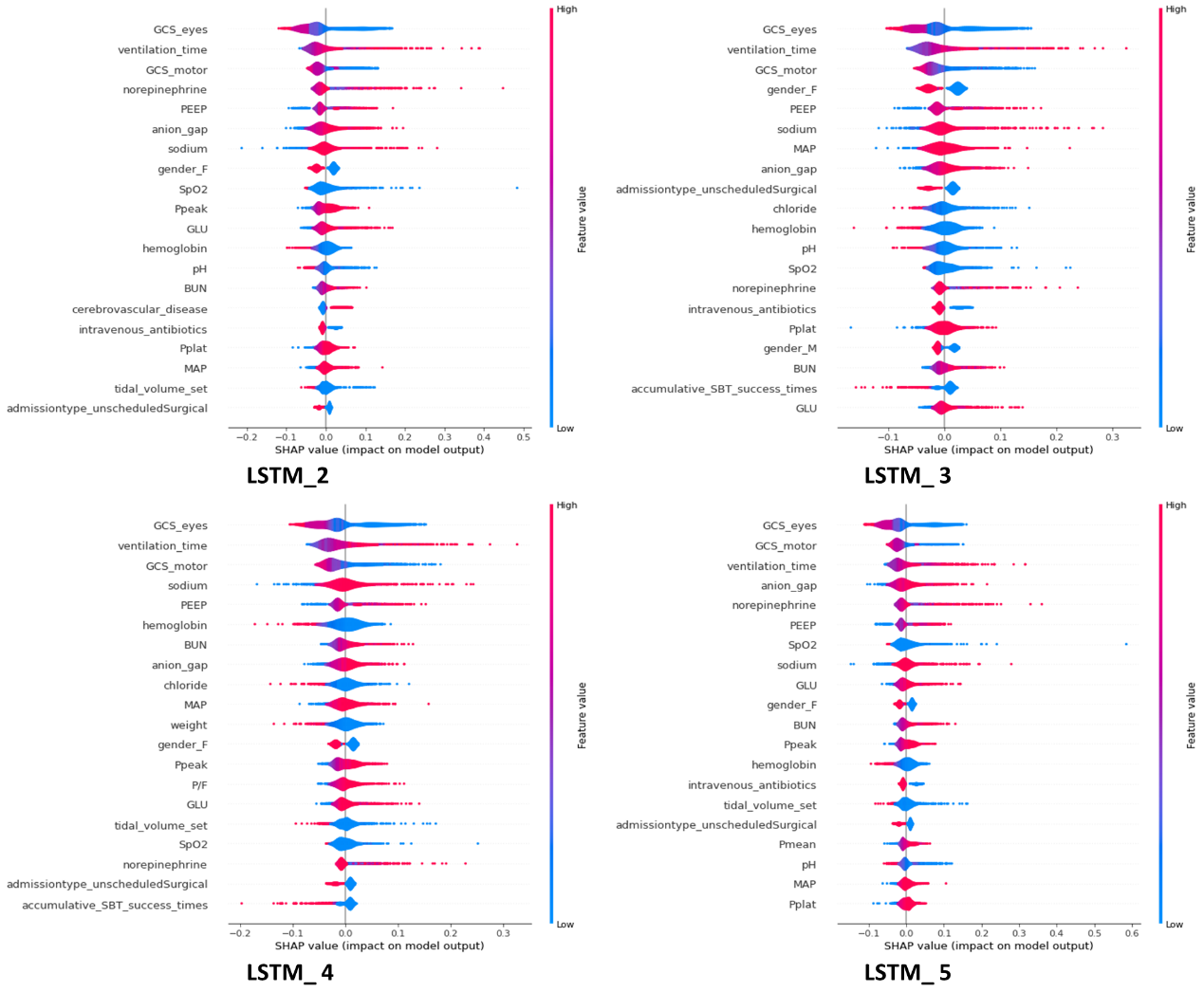


**Supplement Fig 1. The impacts of the top 20 features on predictions of the other four LSTM models.** Abbreviations: GLU glucose, MAP mean arterial pressure, PEEP positive end expiratory pressure, Pmean mean airway pressure, Ppeak peak inspiratory pressure, Pplat airway plateau pressure, P/F PaO2/FiO2.
