# Supplementary material for: Interpretable recurrent neural network models for dynamic prediction of the extubation failure risk in patients with invasive mechanical ventilation in the intensive care unit
Source: BioData Min. 2022 Sep 27;15:21. doi: 10.1186/s13040-022-00309-7 (PMC9513908; doi:10.1186/s13040-022-00309-7)
Supplement: Supplementary file 2 — Additional file 2: SupplementFig 2. Dynamic predictions and SHAP values of a patient by the other four LSTM models. [file 13040_2022_309_MOESM2_ESM.docx]

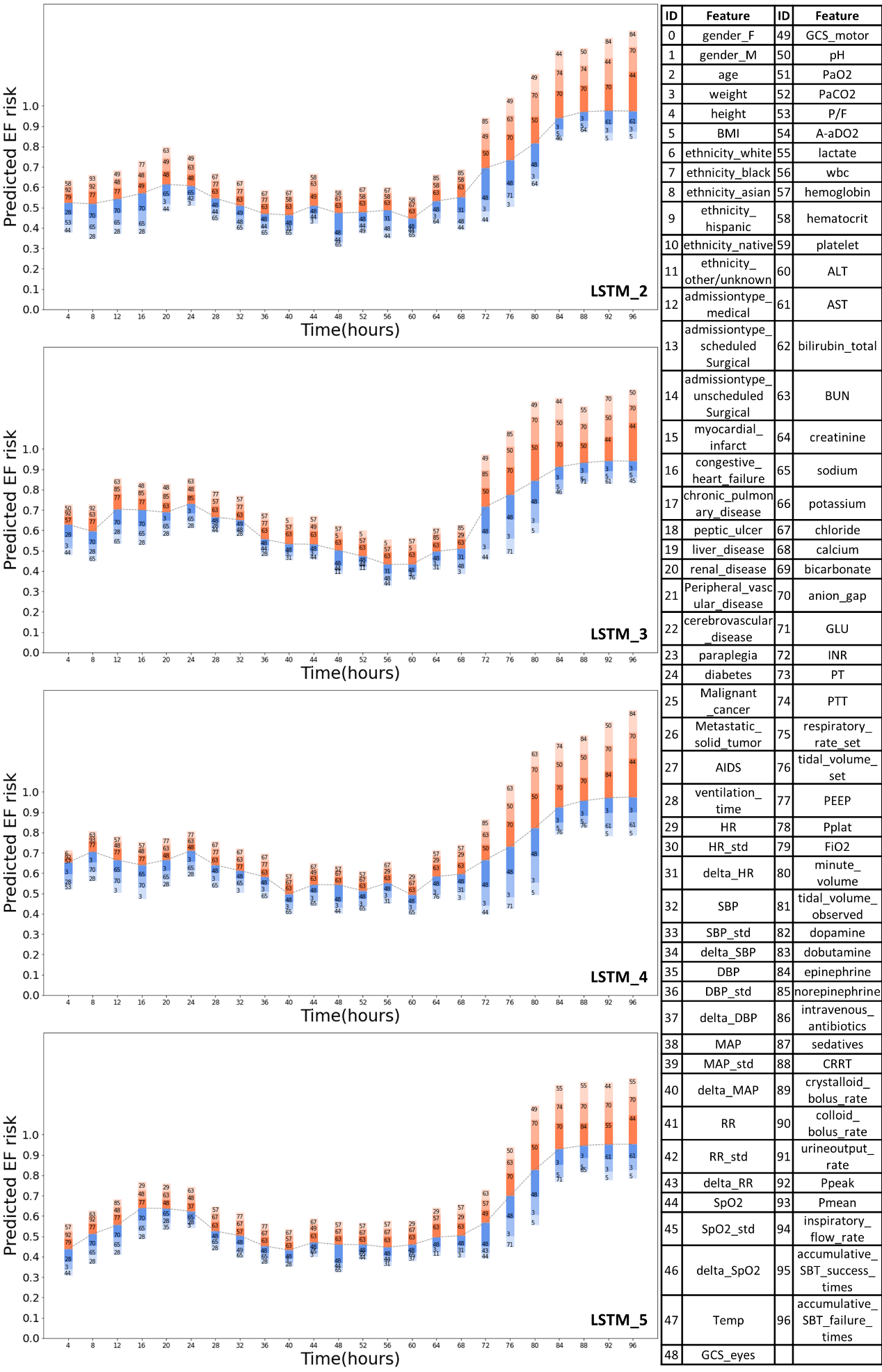


**Supplement Fig 2.** **Dynamic predictions and SHAP values of a patient by the other four LSTM models.** Abbreviations: A-aDO2 alveolar-arterial oxygen difference, BMI body mass index, CRRT continuous renal replacement therapy, DBP diastolic blood pressure, GCS Glasgow Coma Scale, HR heart rate, INR international normalized ratio, MAP mean arterial pressure, PEEP positive end expiratory pressure, Pmean mean airway pressure, Ppeak peak inspiratory pressure, Pplat airway plateau pressure, PT prothrombin time, PTT partial thromboplastin time, P/F PaO2/FiO2, RR respiratory rate, SBP systolic blood pressure, Temp temperature.
