# Supplementary material for: Interpretable recurrent neural network models for dynamic prediction of the extubation failure risk in patients with invasive mechanical ventilation in the intensive care unit
Source: BioData Min. 2022 Sep 27;15:21. doi: 10.1186/s13040-022-00309-7 (PMC9513908; doi:10.1186/s13040-022-00309-7)
Supplement: Supplementary file 3 — Additional file 3: SupplementTable 1. Keywords of the oxygen delivery device and ventilator mode for different ventilation statuses. [file 13040_2022_309_MOESM3_ESM.docx]

**Supplement Table 1** Keywords of the oxygen delivery device and ventilator mode for different ventilation statuses

| Ventilation status | Index keywords in the MIMIC-IV | |
| --- | --- | --- |
|  | Oxygen delivery device | Ventilator mode |
| Invasive ventilation | 'Endotracheal tube', 'Tracheostomy tube' | '(S) CMV', 'APRV', 'APRV/Biphasic+ApnPress', 'APRV/Biphasic+ApnVol', 'APV (cmv)', 'APV (simv)', 'Ambient', 'Apnea Ventilation', 'ASV', 'CMV', 'CMV/ASSIST', 'CMV/ASSIST/AutoFlow', 'CMV/AutoFlow', 'CPAP/PPS', 'CPAP/PSV+Apn TCPL', 'CPAP/PSV+ApnPres', 'CPAP/PSV+ApnVol', 'MMV', 'MMV/AutoFlow', 'MMV/PSV', 'MMV/PSV/AutoFlow', 'P-CMV', 'PCV+', 'PCV+/PSV', 'PCV+Assist', 'PRES/AC', 'PRVC/AC', 'PRVC/SIMV', 'PSV/SBT', 'P-SIMV', 'SIMV', 'SIMV/AutoFlow', 'SIMV/PRES', 'SIMV/PSV', 'SIMV/PSV/AutoFlow', 'SIMV/VOL', 'SYNCHRON MASTER', 'SYNCHRON SLAVE', 'VOL/AC', 'VS' |
| Noninvasive ventilation | 'Bipap mask ', 'CPAP mask ' | 'DuoPaP', 'NIV', 'NIV-ST' |
| High flow oxygen | 'High flow neb', 'High flow nasal cannula' |  |
| Oxygen | 'Nasal cannula', 'Face tent', 'Aerosol-cool', 'Non-rebreather', 'Venti mask ', 'Medium conc mask ', 'T-piece', 'Ultrasonic neb', 'Vapomist', 'Oxymizer' |  |
| None^*^ | None | None |

* ‘None’ status indicates that there is no related record about the oxygen delivery device and ventilation mode during a certain period.
