# Supplementary material for: Interpretable recurrent neural network models for dynamic prediction of the extubation failure risk in patients with invasive mechanical ventilation in the intensive care unit
Source: BioData Min. 2022 Sep 27;15:21. doi: 10.1186/s13040-022-00309-7 (PMC9513908; doi:10.1186/s13040-022-00309-7)
Supplement: Supplementary file 4 — Additional file 4: SupplementTable 2. Overview of the retrieved features. [file 13040_2022_309_MOESM4_ESM.docx]

**Supplement Table 2. Overview of the retrieved features**

| Features | | | | Unit | Normal  ranges^*^ | Reasonable ranges^**^ | Average observing frequency (times/4 hours) | Representations |
| --- | --- | --- | --- | --- | --- | --- | --- | --- |
| Static features | Demographic characteristics | | Gender | — | — | — | — | dummy encoded |
|  |  |  | Age | years | — | — | — | numeric |
|  |  |  | Weight | kg | — | 20-500 | — | numeric |
|  |  |  | Height | m | — | 0.5-2.5 | — | numeric |
|  |  |  | BMI | kg/m^2^ | — | — | — | numeric |
|  |  |  | Ethnicity | — | — | — | — | dummy encoded |
|  |  |  | Admission type | — | — | — | — | dummy encoded |
|  | Comorbidities | | Myocardial infarction | — | — | — | — | 0/1^&^ |
|  |  |  | Congestive heart failure | — | — | — | — | 0/1 |
|  |  |  | Chronic pulmonary disease | — | — | — | — | 0/1 |
|  |  |  | Peptic ulcer disease | — | — | — | — | 0/1 |
|  |  |  | Liver disease | — | — | — | — | 0/1 |
|  |  |  | Renal disease | — | — | — | — | 0/1 |
|  |  |  | Peripheral vascular disease | — | — | — | — | 0/1 |
|  |  |  | Cerebrovascular disease | — | — | — | — | 0/1 |
|  |  |  | Paraplegia | — | — | — | — | 0/1 |
|  |  |  | Diabetes | — | — | — | — | 0/1 |
|  |  |  | Malignant cancer | — | — | — | — | 0/1 |
|  |  |  | Metastatic solid tumor | — | — | — | — | 0/1 |
|  |  |  | AIDS | — | — | — | — | 0/1 |
| Dynamic features | Vital signs | | HR | beats/min | — | 0-300 | 4.57 | mean, std, Δ |
|  |  |  | SBP | mmHg | — | 0-400 | 4.52 | mean, std, Δ |
|  |  |  | DBP | mmHg | — | 0-300 | 4.52 | mean, std, Δ |
|  |  |  | MAP | mmHg | — | 0-300 | 4.77 | mean, std, Δ |
|  |  |  | RR | times/min | — | 0-70 | 4.66 | mean, std, Δ |
|  |  |  | Temp | ℃ | — | 20-50 | 1.43 | mean |
|  |  |  | SpO2 | % | — | 0-100 | 4.50 | mean, std, Δ |
|  | GCS | | GCS eyes | — | — | 1-4 | 1.14 | min |
|  |  |  | GCS motor | — | — | 1-6 | 1.13 | min |
|  | blood biochemistry | Arterial blood gas | pH | — | 7.35-7.45 | 6-8 | 0.62 | mean |
|  |  |  | PaO2 | mmHg | 85-105 | 0-1000 | 0.62 | mean |
|  |  |  | PaCO2 | mmHg | 35-45 | 0-200 | 0.62 | mean |
|  |  |  | P/F | — | — | >0 | 0.62 | mean |
|  |  |  | A-aDO2 | mmHg | — | — | 0.62 | mean |
|  |  |  | Lactate | mmol/L | 0.5-2 | 0-50 | 0.35 | mean |
|  |  |  | Glucose | mg/dL | 70-105 | 0-5000 | 1.23 | mean |
|  |  | Blood routine | WBC | K/uL | 4~10 | 0-1000 | 0.33 | mean |
|  |  |  | Hemoglobin | g/dL | 11.2-15.7 | 0-50 | 0.34 | mean |
|  |  |  | Hematocrit | % | 40-52 | 0-100 | 0.39 | mean |
|  |  |  | Platelet | K/uL | 150-400 | 0-10000 | 0.34 | mean |
|  |  | Liver and renal function | Albumin^#^ | g/dL | 3.5-5.2 | 0-50 | 0.06 | mean |
|  |  |  | Globulin^#^ | g/dL | 2~4 | 0-50 | 0.00 | mean |
|  |  |  | Total protein^#^ | g/dL | 6.4-8.3 | 0-100 | 0.00 | mean |
|  |  |  | Total bilirubin | mg/dL | 0-1.5 | 0-1500 | 0.12 | mean |
|  |  |  | Direct bilirubin^#^ | mg/dL | 0-0.3 | 0-1500 | 0.01 | mean |
|  |  |  | Indirect bilirubin^#^ | mg/dL | — | 0-1500 | 0.01 | mean |
|  |  |  | BUN | mg/dL | 6~20 | 0-300 | 0.37 | mean |
|  |  |  | Creatinine | mg/dL | 0.4-1.1 | 0-150 | 0.37 | mean |
|  |  | Electrolytes | Sodium | mEq/L | 133-145 | 50-200 | 0.40 | mean |
|  |  |  | Potassium | mEq/L | 3.3-5.1 | 0-10 | 0.40 | mean |
|  |  |  | Chloride | mEq/L | 96-108 | 50-200 | 0.40 | mean |
|  |  |  | Calcium | mg/dL | 8.4-10.2 | 0-100 | 0.33 | mean |
|  |  |  | Bicarbonate | mEq/L | 22-32 | 0-100 | 0.38 | mean |
|  |  |  | Anion gap | mEq/L | 8~20 | -50-100 | 0.37 | mean |
|  |  | Coagulation | PT | sec | 9.4-12.5 | 0-150 | 0.25 | mean |
|  |  |  | PTT | sec | 25-36.5 | 0-300 | 0.25 | mean |
|  |  |  | INR | — | 0.9-1.1 | 0-50 | 0.26 | mean |
|  |  |  | D-Dimer^#^ | ng/mL | 0-500 | 0-50000 | 0.00 | mean |
|  |  |  | Fibrinogen^#^ | mg/dL | 150-400 | 0-50000 | 0.06 | mean |
|  |  | Enzymology | ALT | IU/L | 0-40 | 0-100000 | 0.12 | mean |
|  |  |  | AST | IU/L | 0-40 | 0-100000 | 0.12 | mean |
|  |  |  | Amylase^#^ | IU/L | 0-100 | 0-100000 | 0.02 | mean |
|  |  |  | CK^#^ | IU/L | 26-140 | 0-100000 | 0.06 | mean |
|  |  |  | CKMB^#^ | ng/mL | 0-10 | 0-10000 | 0.05 | mean |
|  |  |  | LDH^#^ | IU/L | 94-250 | 0-100000 | 0.07 | mean |
|  |  |  | Troponin T^#^ | ng/mL | 0-0.01 | 0-100 | 0.04 | mean |
|  | Ventilator parameters | | Respiratory rate setting | times/min | — | 0-70 | 0.92 | max |
|  |  |  | Tidal volume setting | ml | — | 0-4000 | 0.88 | max |
|  |  |  | Tidal volume observed | ml | — | 0-4000 | 1.16 | mean |
|  |  |  | Minute volume | L/min | — | 0-200 | 1.1 | mean |
|  |  |  | Inspiratory flow rate | L/min | — | 0-200 | 0.44 | max |
|  |  |  | Ppeak | cmH_2_O | — | 0-200 | 1.09 | max |
|  |  |  | Pmean | cmH_2_O | — | 0-200 | 1.09 | max |
|  |  |  | Pplat | cmH_2_O | — | 0-200 | 0.62 | max |
|  |  |  | PEEP | cmH_2_O | — | 0-200 | 1.19 | max |
|  |  |  | FiO2 | % | — | 21-100 | 1.22 | max |
|  | Vasoactive drugs | | epinephrine | mcg/kg/min | — | >0 | — | max |
|  |  |  | norepinephrine | mcg/kg/min | — | >0 | — | max |
|  |  |  | dopamine | mcg/kg/min | — | >0 | — | max |
|  |  |  | dobutamine | mcg/kg/min | — | >0 | — | max |
|  | Fluid balance | | Crystalloid bolus rate | ml/hour | — | >0 | — | mean |
|  |  |  | Colloid bolus rate | ml/hour | — | >0 | — | mean |
|  |  |  | Urine output rate | ml/hour | — | >0 | — | mean |
|  | Intravenous antibiotics | | | — | — | — | — | 0/1 |
|  | Sedatives | | | — | — | — | — | 0/1 |
|  | CRRT | | | — | — | — | — | 0/1 |
|  | Ventilation time | | | — | — | — | — | numeric |
|  | Accumulative SBT success times | | | — | — | — | — | numeric |
|  | Accumulative SBT failure times | | | — | — | — | — | numeric |

Abbreviations: A-aDO2 alveolar-arterial oxygen difference, BMI body mass index, CRRT continuous renal replacement therapy, DBP diastolic blood pressure, GCS Glasgow Coma Scale, HR heart rate, INR international normalized ratio, MAP mean arterial pressure, PEEP positive end expiratory pressure, Pmean mean airway pressure, Ppeak peak inspiratory pressure, Pplat airway plateau pressure, PT prothrombin time, PTT partial thromboplastin time, P/F PaO2/FiO2, RR respiratory rate, SBP systolic blood pressure, Temp temperature.

* Normal ranges for most blood biochemical items were provided by the MIMIC-IV database.

** Reasonable ranges were used to filter out questionable extreme values.

& 0 represented absence; 1 represented existence.

# Features had an average observing frequency less than 0.1 times/4 hours.
