# Supplementary material for: Interpretable recurrent neural network models for dynamic prediction of the extubation failure risk in patients with invasive mechanical ventilation in the intensive care unit
Source: BioData Min. 2022 Sep 27;15:21. doi: 10.1186/s13040-022-00309-7 (PMC9513908; doi:10.1186/s13040-022-00309-7)
Supplement: Supplementary file 5 — Additional file 5: SupplementTable 3. Dynamic features between the successful and failed extubation group. [file 13040_2022_309_MOESM5_ESM.docx]

**Supplement Table 3** Dynamic features between the successful and failed extubation group

|  | IMV patients（n=8599） | | | | | |
| --- | --- | --- | --- | --- | --- | --- |
|  | Training set (n=6879) | | P value | Test set (n=1720) | | P value |
|  | Successful extubation（n=4790） | Failed extubation（n=2089） |  | Successful extubation（n=1200） | Failed extubation（n=520） |  |
| **Vital signs** |  |  |  |  |  |  |
| HR (beats/min, mean (SD)) | 85.47 (17.36) | 90.98 (20.14) | <0.001 | 85.04 (16.85) | 91.06 (21.12) | <0.001 |
| SBP (mmHg, mean (SD)) | 114.66 (16.02) | 113.42 (17.75) | 0.004 | 115.30 (16.33) | 114.91 (19.08) | 0.665 |
| DBP (mmHg, mean (SD)) | 62.00 (11.35) | 62.18 (12.25) | 0.551 | 62.16 (11.48) | 62.30 (12.85) | 0.821 |
| MAP (mmHg, mean (SD)) | 77.87 (11.67) | 77.11 (12.63) | 0.016 | 78.06 (11.70) | 77.59 (13.42) | 0.466 |
| RR (times/min, mean (SD)) | 18.29 (3.88) | 20.39 (4.51) | <0.001 | 18.12 (3.77) | 20.52 (4.42) | <0.001 |
| Temp (℃, mean (SD)) | 36.71 (0.85) | 36.70 (1.16) | 0.660 | 36.70 (0.86) | 36.64 (1.10) | 0.324 |
| SpO2 (%, mean (SD)) | 98.39 (2.30) | 97.05 (4.67) | <0.001 | 98.48 (2.07) | 97.30 (3.61) | <0.001 |
| **GCS** |  |  |  |  |  |  |
| GCS eyes (median [IQR]) | 1.00 [1.00, 2.00] | 1.00 [1.00, 2.00] | 0.036 | 1.00 [1.00, 2.00] | 1.00 [1.00, 2.00] | 0.146 |
| GCS motor (median [IQR]) | 1.00 [1.00, 5.00] | 1.00 [1.00, 5.00] | 0.104 | 1.00 [1.00, 5.00] | 2.00 [1.00, 5.00] | 0.378 |
| **Blood biochemistry** |  |  |  |  |  |  |
| pH | 7.35 (0.08) | 7.30 (0.11) | <0.001 | 7.35 (0.08) | 7.31 (0.11) | <0.001 |
| PaO2 (mmHg, median [IQR]) | 162.0 [110.0, 228.5] | 128.0 [89.0, 200.] | <0.001 | 156.50 [114.00, 224.00] | 122.00 [86.75, 194.00] | <0.001 |
| PaCO2 (mmHg, mean (SD)) | 41.99 (8.72) | 43.80 (12.41) | <0.001 | 42.08 (9.20) | 42.80 (10.40) | 0.213 |
| P/F (mmHg, median [IQR]) | 248.75 [165.81, 352.00] | 205.00 [126.00, 314.14] | <0.001 | 244.00 [170.96, 342.62] | 192.47 [116.72, 294.08] | <0.001 |
| A-aDO2 (mmHg, median [IQR]) | 255.55 [157.53, 396.96] | 282.55 [169.95, 451.50] | <0.001 | 258.57 [153.77, 398.46] | 302.46 [189.32, 455.88] | <0.001 |
| Lactate (mmol/L, median [IQR]) | 2.10 [1.40, 3.40] | 2.83 [1.60, 5.20] | <0.001 | 2.00 [1.30, 3.10] | 2.70 [1.60, 5.20] | <0.001 |
| Glucose (mg/dL, mean (SD)) | 155.49 (63.47) | 172.15 (81.74) | <0.001 | 153.62 (59.89) | 175.72 (85.30) | <0.001 |
| WBC (K/uL, median [IQR]) | 13.00 [9.20, 17.60] | 13.50 [8.90, 19.60] | 0.038 | 13.00 [9.40, 18.00] | 13.10 [9.33, 18.40] | 0.841 |
| Hemoglobin (g/dL, mean (SD)) | 10.83 (2.02) | 10.86 (2.40) | 0.684 | 10.84 (2.05) | 10.77 (2.34) | 0.625 |
| Hematocrit (%, mean (SD)) | 32.40 (6.04) | 32.95 (7.16) | 0.007 | 32.46 (6.07) | 32.93 (7.05) | 0.232 |
| Platelet (K/uL, median [IQR]) | 166.00 [123.00, 225.00] | 172.00 [114.25, 238.12] | 0.530 | 175.00 [127.00, 236.00] | 175.00 [114.00, 252.00] | 0.350 |
| Total bilirubin (mg/dL, median [IQR]) | 0.80 [0.40, 1.70] | 0.80 [0.50, 1.90] | 0.062 | 0.70 [0.40, 1.40] | 0.80 [0.50, 1.40] | 0.072 |
| BUN (mg/dL, median [IQR]) | 18.00 [13.00, 27.00] | 23.00 [15.00, 39.00] | <0.001 | 18.00 [13.00, 28.00] | 23.00 [15.00, 38.00] | <0.001 |
| Creatinine (mg/dL, median [IQR]) | 0.95 [0.70, 1.30] | 1.20 [0.80, 2.00] | <0.001 | 1.00 [0.70, 1.40] | 1.20 [0.80, 2.00] | <0.001 |
| Sodium (mEq/L, mean (SD)) | 139.00 (4.75) | 139.38 (6.14) | 0.029 | 138.98 (5.12) | 138.97 (5.57) | 0.973 |
| Potassium (mEq/L, mean (SD)) | 4.22 (0.73) | 4.31 (0.86) | 0.001 | 4.24 (0.75) | 4.31 (0.87) | 0.151 |
| Chloride (mEq/L, mean (SD)) | 106.33 (6.13) | 105.13 (7.25) | <0.001 | 106.21 (6.17) | 104.49 (7.01) | <0.001 |
| Calcium (mg/dL, mean (SD)) | 8.12 (1.01) | 8.04 (1.08) | 0.026 | 8.11 (0.95) | 8.12 (1.28) | 0.897 |
| Bicarbonate (mEq/L, mean (SD)) | 22.08 (4.28) | 20.54 (5.51) | <0.001 | 22.18 (4.22) | 20.63 (5.24) | <0.001 |
| Anion gap (mEq/L, mean (SD)) | 14.30 (4.54) | 17.09 (5.92) | <0.001 | 14.26 (4.65) | 17.18 (5.72) | <0.001 |
| PT (sec, median [IQR]) | 14.10 [12.80, 16.00] | 14.80 [13.00, 18.40] | <0.001 | 14.10 [12.80, 15.90] | 14.90 [13.00, 19.45] | <0.001 |
| PTT (sec, median [IQR]) | 31.60 [27.50, 38.30] | 33.80 [28.00, 46.30] | <0.001 | 31.12 [27.10, 37.70] | 33.20 [27.70, 45.52] | 0.004 |
| INR (-,median [IQR]) | 1.30 [1.20, 1.40] | 1.30 [1.20, 1.70] | <0.001 | 1.30 [1.20, 1.45] | 1.35 [1.20, 1.80] | <0.001 |
| ALT (IU/L, median [IQR]) | 37.50 [19.00, 115.00] | 55.00 [26.00, 183.75] | <0.001 | 31.00 [16.00, 110.00] | 42.00 [20.00, 142.00] | 0.020 |
| AST (IU/L, median [IQR]) | 58.00 [31.00, 186.00] | 98.50 [43.00, 348.50] | <0.001 | 48.00 [25.50, 179.50] | 77.00 [38.00, 251.00] | 0.001 |
| **Ventilator parameters** |  |  |  |  |  |  |
| Respiratory rate setting (times/min, mean (SD)) | 17.95 (4.27) | 20.01 (5.20) | <0.001 | 17.74 (3.98) | 20.11 (5.31) | <0.001 |
| Tidal volume setting (ml, mean (SD)) | 476.73 (70.97) | 461.41 (74.24) | <0.001 | 473.24 (70.14) | 461.69 (70.51) | 0.002 |
| Tidal volume observed (ml, mean (SD)) | 488.64 (101.01) | 475.94 (109.44) | <0.001 | 483.93 (97.28) | 474.29 (98.04) | 0.061 |
| Minute volume (L/min, mean (SD)) | 8.69 (2.27) | 9.50 (2.84) | <0.001 | 8.53 (2.25) | 9.51 (2.61) | <0.001 |
| Inspiratory flow rate (L/min, mean (SD)) | 43.69 (9.96) | 45.08 (10.84) | 0.004 | 42.85 (10.14) | 44.88 (10.38) | 0.034 |
| Ppeak (cmH2O, mean (SD)) | 24.06 (6.32) | 25.84 (7.17) | <0.001 | 23.82 (6.06) | 26.27 (7.11) | <0.001 |
| Pmean (cmH2O, mean (SD)) | 10.96 (3.73) | 12.35 (4.31) | <0.001 | 10.65 (3.18) | 12.60 (4.38) | <0.001 |
| Pplat (cmH2O, mean (SD)) | 18.82 (4.91) | 20.79 (5.69) | <0.001 | 18.62 (4.65) | 20.66 (5.49) | <0.001 |
| PEEP (cmH2O, mean (SD)) | 7.00 (3.06) | 7.88 (3.84) | <0.001 | 6.75 (2.77) | 8.11 (3.96) | <0.001 |
| FiO2 (%, mean (SD)) | 78.52 (25.28) | 77.24 (25.06) | 0.054 | 76.64 (25.83) | 78.64 (24.41) | 0.136 |
| **Vasoactive drugs*** |  |  |  |  |  |  |
| Epinephrine n (%) | 551 (11.5) | 212 (10.1) | 0.109 | 144 (12.0) | 51 (9.8) | 0.217 |
| Norepinephrine n (%) | 1178 (24.6) | 867 (41.5) | <0.001 | 280 (23.3) | 227 (43.7) | <0.001 |
| Dopamine n (%) | 123 (2.6) | 107 (5.1) | <0.001 | 32 (2.7) | 24 (4.6) | 0.052 |
| Dobutamine n (%) | 45 (0.9) | 39 (1.9) | 0.002 | 7 (0.6) | 8 (1.5) | <0.001 |
| **Fluid balance** |  |  |  |  |  |  |
| Crystalloid bolus rate (ml/hour, median [IQR]) | 0.00 [0.00, 250.00] | 0.00 [0.00, 250.00] | <0.001 | 0.00 [0.00, 291.31] | 0.00 [0.00, 250.00] | 0.026 |
| Colloid bolus rate (ml/hour, median [IQR]) | 0.00 [0.00, 0.00] | 0.00 [0.00, 0.00] | 0.010 | 0.00 [0.00, 0.00] | 0.00 [0.00, 0.00] | 0.100 |
| Urine output rate (ml/hour, median [IQR]) | 72.50 [36.25, 132.50] | 47.50 [17.50, 100.00] | <0.001 | 71.25 [33.75, 129.62] | 50.00 [15.94, 105.00] | 0.094 |
| **Intravenous antibiotics n (%)** | 3158 (65.9) | 1296 (62.0) | 0.002 | 767 (63.9) | 341 (65.6) | 0.545 |
| **Sedatives n (%)** | 4336 (90.5) | 1754 (84.0) | <0.001 | 1082 (90.2) | 437 (84.0) | <0.001 |
| **CRRT n (%)** | 32 (0.7) | 37 (1.8) | <0.001 | 12 (1.0) | 5 (1.0) | 0.848 |
| **Accumulative SBT success times (median [IQR])** | 0.00 [0.00, 0.00] | 0.00 [0.00, 0.00] | 0.989 | 0.00 [0.00, 0.00] | 0.00 [0.00, 0.00] | 0.773 |
| **Accumulative SBT failure times (median [IQR])** | 0.00 [0.00, 0.00] | 0.00 [0.00, 0.00] | 0.960 | 0.00 [0.00, 0.00] | 0.00 [0.00, 0.00] | 0.943 |

Abbreviations: A-aDO2 alveolar-arterial oxygen difference, CRRT continuous renal replacement therapy, DBP diastolic blood pressure, GCS Glasgow Coma Scale, HR heart rate, INR international normalized ratio, MAP mean arterial pressure, PEEP positive end expiratory pressure, Pmean mean airway pressure, Ppeak peak inspiratory pressure, Pplat airway plateau pressure, PT prothrombin time, PTT partial thromboplastin time, P/F PaO2/FiO2, RR respiratory rate, SBP systolic blood pressure, Temp temperature.

* The dose of vasoactive drug was not presented since data is too sparse for statistical analysis. Instead, usage of vasoactive drug was presented as categorical variable in this table. But the dose of vasoactive drug was still used for model development.
