# Supplementary material for: Interpretable recurrent neural network models for dynamic prediction of the extubation failure risk in patients with invasive mechanical ventilation in the intensive care unit
Source: BioData Min. 2022 Sep 27;15:21. doi: 10.1186/s13040-022-00309-7 (PMC9513908; doi:10.1186/s13040-022-00309-7)
Supplement: Supplementary file 6 — Additional file 6: SupplementTable 4. Optimized hyperparameters and AUROCs of the models in five-fold CV. [file 13040_2022_309_MOESM6_ESM.docx]

**Supplement Table 4** Optimized hyperparameters and AUROCs of the models in five-fold CV

|  | Major optimized hyperparameters* | 5-fold CV on the training set  Mean AUROC [95%CI] |
| --- | --- | --- |
| LSTM | hidden_layers=1; hidden_size=20; dropout=0.5; learning rate=0.001; activation function=’ReLU’; batch_size=32 | 0.814 [0.802, 0.826] |
| GRU |  | 0.817 [0.803, 0.831] |
| Lasso LR | penalty='l1'; solver='liblinear'; C=1.0; | 0.800 [0.787, 0.813] |
| SVM | kernel='rbf'; C=0.8; gamma=0.005 | 0.811 [0.800, 0.821] |
| MLP | hidden_layer_sizes=(3, 3); activation function ='relu'; solver='adam'; learning_rate_init=0.001; batch_size='auto' | 0.801 [0.788, 0.815] |
| RF | n_estimators=200; max_depth=6; min_samples_split=2; min_samples_leaf=2; max_features='sqrt'; | 0.815 [0.798, 0.831] |
| XGB | n_estimators=200; learning_rate=0.1; colsample_bytree=0.6; subsample=0.6; max_depth=6; min_child_weight=1; gamma=0.2 | 0.814 [0.803, 0.824] |

Abbreviations: LSTM long short-term memory, GRU gated recurrent unit, LR logistic regression, SVM support vector machine, MLP multi-layer perceptron, RF random forest, XGB extreme gradient boosting, CV cross validation.

* The optimized hyperparameters were searched from the following settings:

LSTM/GRU: hidden layers of LSTM/GRU (1, 2, 3, 4), hidden size (5, 10, 15, 20, 25), dropout (0, 0.25, 0.5, 0.75), learning rate (0.01, 0.001), activation function (sigmoid, ReLU);

Lasso LR: C (0.8, 1.0, 1.2);

SVM: C (0.8, 1.0, 1.2), gamma (0.001, 0.003,0.005, 0.007);

MLP: hidden_layer_sizes ((1,3), (3,3), (4,4), (5,5)); activation function (sigmoid, ReLU), learning_rate_init (0.01, 0.001);

RF: n_estimators (100, 200, 400), max_depth (4, 6), min_samples_split (2, 4), min_samples_leaf (2, 4);

XGB: n_estimators (100, 200, 400), learning_rate (0.01,0.1), colsample_bytree (0.4, 0.6, 0.8), subsample (0.4, 0.6, 0.8), max_depth (4, 6), min_child_weight (0.5, 1.0), gamma (0.2, 0.4).
